# The PAS domain of the polarly localized histidine kinase FlrB in *Vibrio cholerae* controls class III flagellar transcription and contributes to intestinal colonization

**DOI:** 10.1128/mbio.02379-25

**Published:** 2025-09-22

**Authors:** Venus Stanton, Bradley Himes, Adrian Mejia-Santana, Mark Eppinger, Jesus Romo, Jiawei Xing, Igor B. Zhulin, Hong Cai, Yufeng Wang, Nicole l. Inniss, George Minasov, Karla J. F. Satchell, Karl E. Klose

**Affiliations:** 1 South Texas Center for Emerging Infectious Diseases and Department of Molecular Microbiology and Immunology, University of Texas at San Antonio 12346, San Antonio, Texas, USA; 2 Department of Microbiology, Translational Data Analytics Institute, The Ohio State University 2647, Columbus, Ohio, USA; 3 Department of Microbiology-Immunology and Center for Structural Biology of Infectious Diseases, Northwestern University Feinberg School of Medicine 12244, Chicago, Illinois, USA; University of Georgia, Athens, Georgia, USA

**Keywords:** cholera, transcriptional regulation, signal transduction, flagellar motility, pathogenesis, subcellular localization

## Abstract

*Vibrio cholerae* motility is mediated by a single polar flagellum, composed of four flagellin subunits (FlaABCD) in the filament; however, only FlaA is required for motility. Class III flagellar genes, which include *flaA*, are controlled by the two-component FlrBC system. FlrB is a histidine kinase that phosphorylates FlrC, which activates Class III promoters. The signal(s) that control phosphotransfer between FlrB and FlrC are unknown. A *V. cholerae* strain lacking the “non-essential” flagellin genes (Δ*flaCEDB*) is non-motile. Selection for spontaneous motile strains resulted in mutations localized to a Per-Arnt-Sim (PAS) domain in the FlrB N-terminus, including the mutation L36F. The X-ray crystal structure of FlrB revealed an asymmetric dimer with a unique fold of the PAS domain. Transcriptome analysis showed that class III transcription is increased with the addition of the L36F PAS mutation to FlrB, while class III gene transcription was eliminated with a mutation at the site of phosphorylation (H135N). H135N prevents phosphorylation of purified FlrB, whereas L36F increases phosphorylation, indicating these mutations represent “off” and “on” forms of FlrB. FlrB localizes to the *V. cholerae* cell pole, and localization is dependent on the flagellar polar targeting protein FlhF. *V. cholerae* strains containing either “off” or “on” forms of FlrB were defective for intestinal colonization in infant mice (10- to 40-fold defect). Our results demonstrate that the PAS domain controls FlrB activity and class III flagellar gene expression, and FlrB must switch between inactive and active forms in order for *V. cholerae* to successfully colonize the intestine.

**IMPORTANCE:**

*Vibrio cholerae* causes the severe diarrheal disease cholera when it colonizes the human intestine. The bacteria are able to swim due to a polar flagellum, and motility is linked to disease, as well as environmental persistence. This study demonstrates that FlrB, a key regulatory protein, localizes to the cell pole and controls flagellar gene transcription via a PAS domain that regulates autophosphorylation. The ability of FlrB to switch between active and inactive forms is critical for motility, as well as intestinal colonization, emphasizing the importance of *V. cholerae* swimming for its ability to cause disease.

## INTRODUCTION

*Vibrio cholerae* (*Vc*) causes the potentially fatal diarrheal disease cholera when it colonizes the human intestine and expresses cholera toxin. *Vc*, like most *Vibrio* spp., has a single polar flagellum that facilitates motility (1). Motility and chemotaxis have been linked to intestinal colonization and disease, and connection(s) of motility to pathogenesis continue to be discovered (2–5).

**Fig 1:**
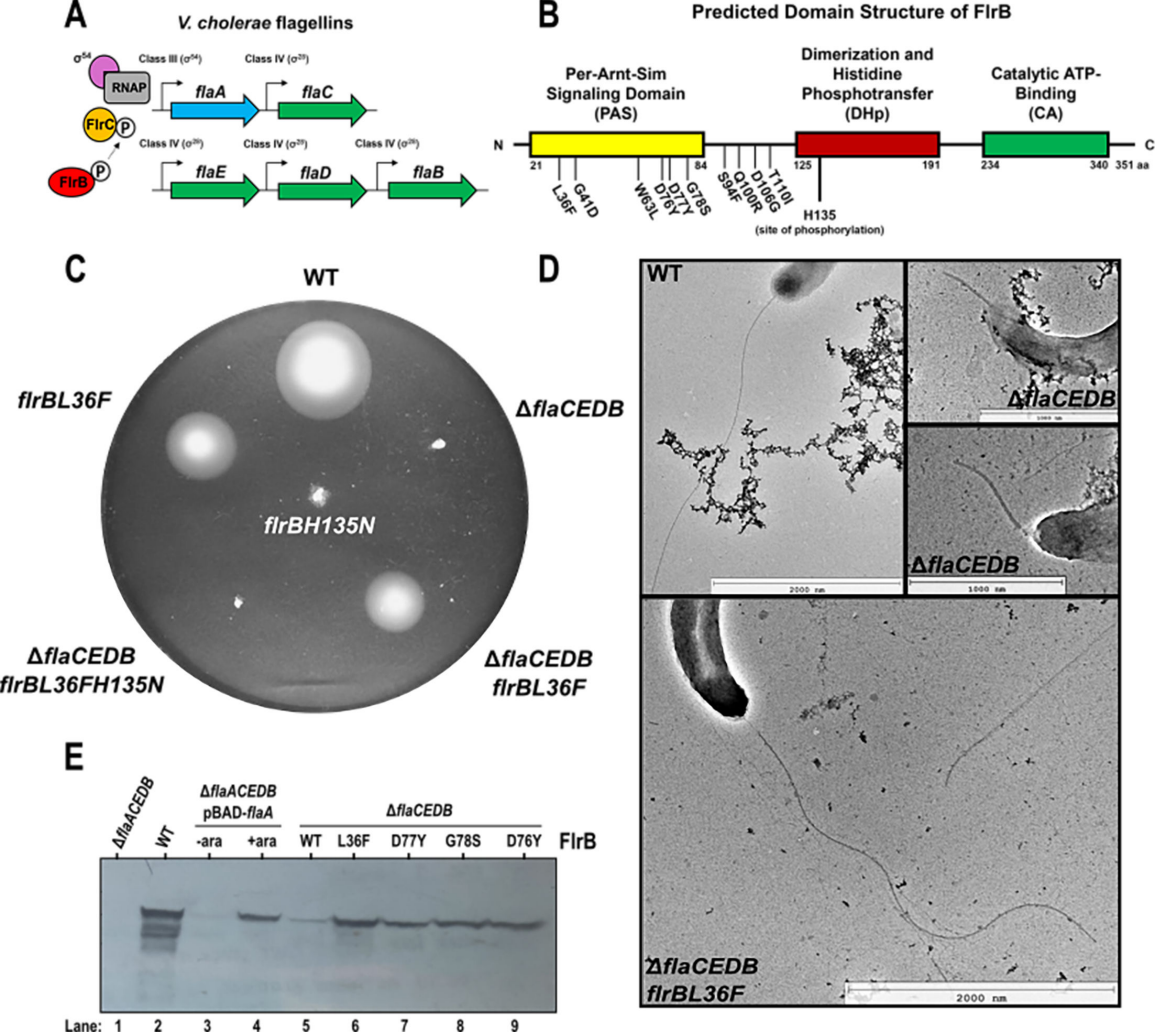
FlrB-PAS mutations restore motility to non-motile *Vc*Δ*flaCEDB*. (**A**) Schematic of the five flagellin genes. *flaA* (blue) is a FlrC- and σ^54^-dependent class III gene, while *flaB*, *flaC*, *flaD*, and *flaE* (green) are σ^28^-dependent class IV genes. FlrB (red) autophosphorylates and transfers the phosphate to FlrC (orange). FlrC-P, along with σ^54^-RNA polymerase holoenzyme, activates class III flagellar promoters. (**B**) Predicted domains of FlrB. Mutations in or near the PAS domain are denoted. The site of phosphorylation (H135) is indicated. (**C**) Motility of *Vc flrB* mutant strains. *Vc* strains were inoculated into motility agar at 30°C; motility is visualized by swarm diameter. Strains shown are KKV598 (wild type), KKV2530 (Δ*flaCEDB*), KKV4091 (Δ*flaCEDB flrBL36F*), KKV4090 (Δ*flaCEDB flrBL36F H135N*), KKV3288 (*flrBL36F*), and KKV3415 (*flrBH135N*). (**D**) Transmission electron microscopy of *Vc* strains KKV598 (wild type), KKV2530 (Δ*flaCEDB*), and KKV2579 (Δ*flaCEDB flrBL36F*). (**E**) FlrB-PAS mutations induce elevated levels of FlaA. Western immunoblot with αflagellin antibody of the following *Vc* strains: Lanes: 1, KKV2431 (Δ*flaACEDB*); 2, KKV598 (WT); 3 and 4, KKV2431 (Δ*flaACEDB*) carrying pKEK1948 (pBAD-*flaA*) grown without (lane 3) or with (lane 4) 0.2% arabinose; 5, KKV2530 (Δ*flaCEDB*); 6, KKV2579 (Δ*flaCEDB*; *flrBL36F*); 7, KKV2575 (Δ*flaCEDB*; *flrBD77Y*); 8, KKV3174 (Δ*flaCEDB*; *flrBG78S*); 9, KKV4089 (Δ*flaCEDB*; *flrBD76Y*).

Flagellins are the structural proteins that make up the flagellar filament. *Vc* expresses five distinct flagellins, FlaABCDE (6) (Fig. 1A). A *flaA* mutant is non-flagellated and non-motile, whereas single mutations in the other four flagellin genes have no effect on flagellar synthesis (6, 7). Moreover, overexpression of FlaA, but not the other flagellins, in a Δ*flaABCDE* strain provides motility, indicating that FlaA is essential and sufficient for filament synthesis and motility (7). The FlaB, FlaC, and FlaD flagellins are found within the filament (8, 9), and in the absence of FlaA, these flagellins are secreted (10). In contrast, FlaE appears to be a biofilm matrix protein (9). Cryoelectron microscopy demonstrated FlaA at the base of the filament, providing a “link” between junction proteins FlgK/FlgL and the other flagellins, illuminating the dependence of filament formation on FlaA (W. Guo, S. Zhang, J. H. Park, V. Stanton, M. Asp, H. Herrera, J.-S.B.Tai, J. Yue, J. Wang, S. Wu, J. Yan, K. E. Klose, F. Yildiz, and J. Liu, unpublished data). *flaA* transcription is activated by the response regulator FlrC, which binds to an enhancer site to activate σ^54^-dependent transcription (6, 11) (Fig. 1A). FlrC is phosphorylated at D54 within its receiver domain by histidine kinase FlrB (12), and the FlrC central domain forms a heptamer with ATPase activity that catalyzes σ^54^-dependent transcription (13). The *Vc* flagellar genes are controlled by a four-tiered transcription hierarchy (1, 14, 15). The class I gene, *flrA*, activates transcription of class II genes, which encode components of the flagellar secretion apparatus, along with FlrBC, σ^28^, and FlhFG that control the number and location of the flagellum (16). Phospho-FlrC activates transcription of class III genes, which, along with *flaA*, also include components of the rod-hook structure (14, 15). Finally, secretion of the anti-sigma factor FlgM allows σ^28^-RNA polymerase to activate class IV genes, which include the *flaCEDB* flagellins (6, 14, 17), along with motor and chemotaxis genes. *Vc* strains with mutations in the export apparatus are defective for FlrC-dependent transcription (18), but it is unclear what signal(s) governs the class II to class III switch.

In the present study, we have identified mutations within the FlrB PAS domain that increase FlrB autophosphorylation and FlrC-dependent gene transcription, suggesting that these mutations push FlrB into an “on” state. In contrast, alteration of the site of autophosphorylation (H135N) locks FlrB into an “off” state, preventing FlrB-dependent gene transcription. *Vc* strains with FlrB locked “on” or “off” are defective for intestinal colonization, illuminating the need for FlrB to switch between active and inactive states for *Vc* virulence. FlrB localizes to the flagellar pole in a FlhF-dependent manner, suggesting that localization may be associated with FlrB activity.

## RESULTS

### Δ*flaCEDB Vc* is non-motile: mutations in FlrB restore motility

We have previously shown that a *flaA* mutant *Vc* strain is non-motile (6), whereas a strain lacking all five flagellins (Δ*flaACEDB*) regains motility when FlaA is overexpressed (7), demonstrating that FlaA is necessary and sufficient for motility. Curiously, *Vc* lacking the four “non-essential” flagellins (Δ*flaCEDB*) is non-motile, in comparison to wild type (Fig. 1C). Prolonged incubation of Δ*flaCEDB Vc* in motility agar led to the recovery of spontaneous motile mutants. Two motile mutant Δ*flaCEDB* strains were subjected to whole-genome sequencing, which revealed that each had a single nucleotide change in *flrB*, which encodes the histidine kinase that phosphorylates FlrC. The mutations (L36F and G41D) lie within the Per-Arnt-Sim (PAS) domain within the N-terminus (Fig. 1B). Five additional spontaneous motile Δ*flaCEDB* strains were isolated and subjected to focused sequencing of *flrB*, which revealed mutations that cluster into the FlrB PAS domain (W63L, D77Y, S94F, Q100R, and T110I; Fig. 1B; Fig. S1). Three motile mutants were also obtained in El Tor biotype Δ*flaCEDB* and subjected to focused *flrB* sequencing, which revealed mutations within the FlrB N-terminus (D76Y, G78S, and D106G; Fig. 1B; Fig. S1), all clustered within or close to the PAS domain.

Two of the *flrB* mutations (L36F and D76Y) were re-introduced into the genome of *Vc* Δ*flaCEDB*, which confirmed that these PAS mutations were responsible for the gain in motility (Fig. 1C; Fig. S2). The *flrBL36F* mutation was also introduced into the genome of an otherwise wild-type strain (Fig. 1C), and this strain showed reduced motility (perhaps due to excess class III gene expression, see below). A mutation at the predicted site of autophosphorylation, H135N, was introduced into the wild-type *Vc* genome, and this strain became non-motile (Fig. 1C), consistent with phospho-FlrB being required for *Vc* motility. We demonstrate below that H135 is the site of phosphorylation. Introduction of the H135N mutation into *flrBL36F* Δ*flaCEDB Vc* also results in non-motility, indicating that L36F requires FlrB phosphorylation to facilitate motility of Δ*flaCEDB* (i.e., L36F does not “bypass” the requirement for FlrB phosphorylation; Fig. 1C). Cumulatively, these results demonstrate that mutations in FlrB PAS allow motility of Δ*flaCEDB Vc*, and the mutations require phospho-FlrB.

### FlrB PAS mutation restores flagellar synthesis to Δ*flaCEDB Vc*

Transmission electron microscopy was performed on Δ*flaCEDB Vc* cells to observe the flagellar filament. Non-motile Δ*flaCEDB* cells, which only encode *flaA*, express a truncated filament, in contrast to wild-type *Vc*, which expresses a full-length filament (Fig. 1D). Introduction of *flrBL36F* into Δ*flaCEDB* restored filament synthesis in these cells (Fig. 1D), demonstrating that the PAS mutation restores motility to Δ*flaCEDB* by restoring filament synthesis.

Western immunoblot analysis was performed on whole cell lysates to quantitate FlaA expression in the various *Vc* strains, using anti-flagellin antisera (Fig. 1E). Equivalent amounts of protein were loaded into each well. *Vc* lacking all flagellins (Δ*flaACEDB*; lane 1) showed no flagellin expression, in contrast to wild-type *Vc* (lane 2), in which several flagellin bands were visible. The Δ*flaACEDB* strain carrying a plasmid expressing FlaA from the pBAD promoter, grown in the absence/presence of arabinose (lanes 3 and 4), shows FlaA only in the presence of arabinose. Δ*flaCEDB Vc* expresses low levels of FlaA (lane 5), and the introduction of the *flrB* mutations L36F (lane 6), D77Y (lane 7), G78S (lane 8), or D76Y (lane 9) into Δ*flaCEDB* results in higher levels of FlaA in these strains. These results indicate that the PAS mutations result in higher levels of FlaA in Δ*flaCEDB Vc*.

### H135 is the site of FlrB phosphorylation, and L36F increases phospho-FlrB

**Fig 2:**
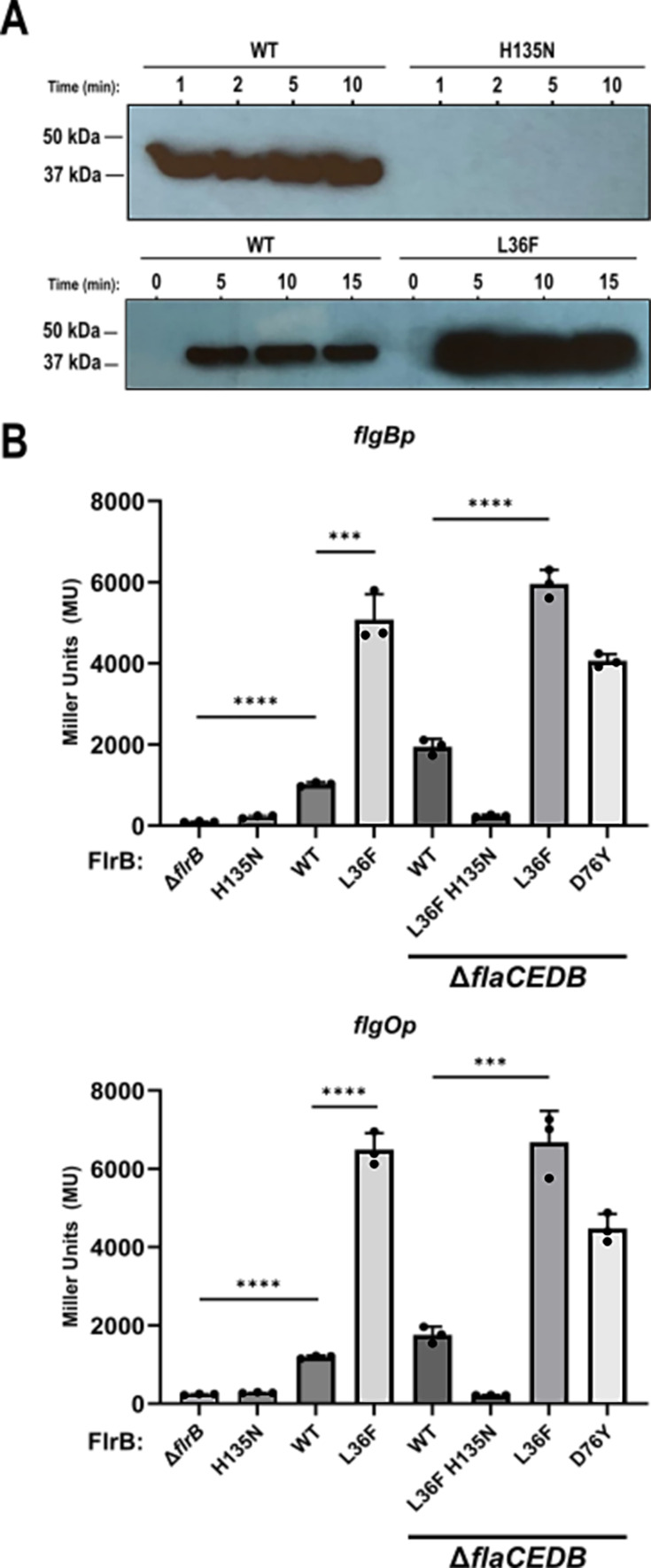
Increased autophosphorylation and increased class III transcription by FlrB PAS mutations. (**A**) H135N prevents FlrB autophosphorylation, and L36F increases FlrB autophosphorylation. Western immunoblot using anti-para-nitro-benzo-mesylate (PNBM) antibody to detect levels of phosphorylation of FlrB^H135N^ (top right) and FlrB^L36F^ (bottom right) compared to native FlrB. (**B**) FlrB-PAS mutations increase transcription of class III flagellar genes. *Vc* strains KKV176 (Δ*flrB*), KKV3415 (*flrBH135N*), KKV598 (wild type), KKV3288 (*flrBL36F*), KKV2530 (Δ*flaCEDB*), KKV4090 (Δ*flaCEDB flrBL36F H135N*), and KKV4089 (Δ*flaCEDB flrBD76Y*) carrying pKEK332 (*flgB*p*-lacZ*, top) or pKEK782 (*flgO*p-*lacZ*, bottom) were assayed for β-galactosidase activity (see Materials and Methods) (unpaired, two-tailed Student’s *t*-test: ***P ≤ 0.001; ****P ≤ 0.0001).

FlrB is a histidine kinase with a conserved dimerization and histidine phosphotransfer domain (DHp; aa 125-191; Fig. 1B) and catalytic and ATP binding domain (CA; aa 234–340). FlrB is predicted to autophosphorylate at the conserved histidine residue H135 (19). We performed a phosphorylation assay with purified wild-type and H135N FlrB, using ATPγS and subsequent detection of thiophosphohistidine ([20]; Fig. 2A). Wild-type FlrB autophosphorylates rapidly in the presence of ATPγS. In contrast, no phospho-FlrB H135N could be detected, consistent with H135 being the site of autophosphorylation (Fig. 2A top).

The phosphorylation assay was also performed with purified wild-type and L36F FlrB (Fig. 2A bottom). Interestingly, more phospho-FlrB L36F could be detected at all time points, indicating that L36F causes an increase in FlrB autophosphorylation. These results are consistent with the increased class III gene transcription of *flrBL36F Vc* strains (below).

### FlrB PAS mutations increase class III gene transcription

The results above indicate that FlrB PAS mutations cause higher levels of autophosphorylation, thus increasing phosphotransfer to FlrC and higher levels of class III transcription. To verify that class III transcription is upregulated in strains containing FlrB PAS mutations, we measured transcription of two class III flagellar promoters, *flgB*p and *flgO*p, fused to a *lacZ* reporter in various *Vc* strains (14, 21) (Fig. 2B). The patterns of transcription of the *flgB* and *flgO* promoters were similar. First, comparing transcription in wild type vs strains either without FlrB (Δ*flrB*) or with FlrB unable to be phosphorylated (*flrBH135N*) demonstrated that transcription at these promoters requires phospho-FlrB. Second, transcription of both promoters was elevated in Δ*flaCEDB* compared to wild type, indicating that the lack of motility of the Δ*flaCEDB* strain is not due to downregulation of class III transcription. Third, the introduction of the *flrBL36F* mutation caused a significant three- to sixfold increase in transcription at *flgB*p and *flgO*p in either Δ*flaCEDB* or wild-type backgrounds, demonstrating that the L36F PAS mutation enhances FlrB-dependent transcription. Fourth, increased transcription due to the L36F mutation requires phosphorylation of FlrB because transcription was eliminated in the *flrBL36F H135N* strain. Fifth, this phenomenon is not restricted to the L36F mutation because the introduction of another PAS domain mutation, D76Y, into the Δ*flaCEDB* background also caused a two- to threefold increase in transcription at *flgB*p and *flgO*p.

### Transcriptome identifies FlrB-dependent (class III) transcription patterns

**Fig 3:**
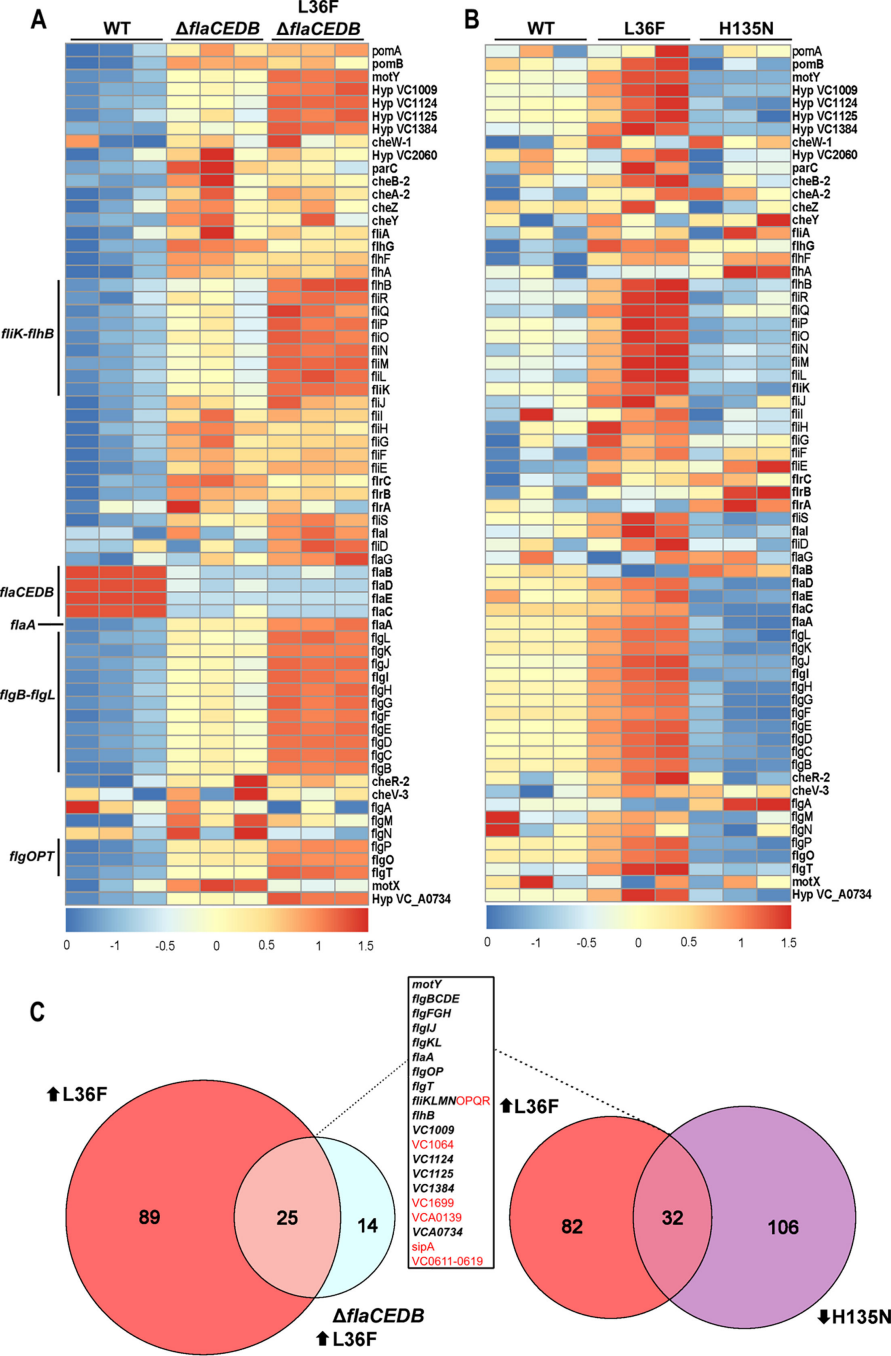
Cluster analysis of flagellar gene transcription in FlrB mutant strains. RNAseq was performed on the indicated strains (three biological replicates each). Transcription of each of the *Vc* flagellar genes is shown in a heat map, with the relative transcription of each gene among all samples shown. Strains used: KKV598 (wild type), KKV2530 (Δ*flaCEDB*), KKV2579 (Δ*flaCEDB flrBL36F*), KKV3288 (*flrBL36F*), and KKV3415 (*flrBH135N*). Actual values in File S1. (**A**) Comparison of flagellar gene transcription in wild type, Δ*flaCEDB*, and Δ*flaCEDB flrBL36F*. (**B**) Comparison of flagellar gene transcription in wild type, *flrBL36F*, and *flrBH135N*. (**C**) Overlap of genes upregulated by L36F and downregulated by H135N reveals core FlrBC-regulated genes. Venn diagrams indicate genes upregulated in KKV2579 (Δ*flaCEDB flrBL36F*) and KKV3288 (*flrBL36F*), in comparison to respective parent strains, as well as genes upregulated in KKV3288 (*flrBL36F*) and downregulated in KKV3415 (*flrBH135N*). Genes common to overlap in both Venn diagrams and/or previously identified as FlrC-dependent are shown in black, and genes significantly differentially expressed in only one overlap are shown in red.

We performed RNAseq on wild type, Δ*flaCEDB*, and Δ*flaCEDB flrBL36F Vc* (three independent replicates each). The transcriptome of Δ*flaCEDB* showed 235 genes upregulated and 124 genes downregulated, compared to the wild-type strain (File S1; Fig. 3A). To avoid confusion, the corresponding gene number in *Vc* strain N16961 is used here (22) to follow convention in the field; gene designations from the parent strain are in File S1. Class III flagellar gene transcription was upregulated in Δ*flaCEDB*, confirming that the non-motile phenotype is not due to low class III expression.

When comparing the transcriptomes of Δ*flaCEDB* to Δ*flaCEDB flrBL36F*, only 28 genes (excluding 11 rRNA/tRNAs) were upregulated by L36F (Fig. 3A and C; File S1). Twenty-two of these genes had been previously identified as FlrC-dependent genes (6, 14, 15, 23), while the remaining six genes encode proteins with various predicted functions.

A heat map of the transcription patterns of the flagellar genes in wild-type, Δ*flaCEDB*, and Δ*flaCEDB flrBL36F* strains is shown (Fig. 3A). Colors indicate relative expression of each gene across the three strains compared to each other; the actual values are in File S1. The transcription pattern of Δ*flaCEDB* with the addition of the *flrBL36F* mutation demonstrates upregulation of class III flagellar gene clusters, consistent with the L36F mutation causing higher levels of FlrC-dependent transcription.

To determine if the FlrB L36F “Up” mutation was also able to increase class III transcription in a wild-type background, we performed RNAseq analyses on *flrBL36F*, as well as *flrBH135N*, which prevents autophosphorylation, and compared the transcriptomes to wild type (three independent replicates each). The transcriptome of *flrBL36F* showed 114 genes upregulated and 54 genes downregulated, in comparison to wild type (Fig. 3B; File S1). Importantly, the 19 upregulated FlrB-dependent flagellar genes identified in the Δ*flaCEDB* background (Fig. 3A) were also upregulated in the wild-type background. This analysis also identified the *fliOPQRflhB* and *flgOP* genes as upregulated by FlrB L36F. Additional genes upregulated in *flrBL36F* were also upregulated in Δ*flaCEDB flrBL36F* (VC1009, VC1124, VC1125, VCA0734, and VC1699), as well as genes involved in chitin utilization (24, 25) and sialic acid metabolism (26). Interestingly, 18 upregulated genes are within a prophage exclusive to classical O1. Two identical copies of the prophage are integrated within the O395 genome.

The transcriptome of *flrBH135N* showed 138 genes downregulated and 132 genes upregulated, in comparison to wild type (Fig. 3B; File S1). The *flgBCDEFGHIJKL*, *flgOP*, *fliK*, *flaA*, and *motY* genes were downregulated. VC1009, VC1124, VC1125, and VCA0734 were also downregulated. Additional genes downregulated in *flrBH135N* included critical virulence genes (*tcpARDSTEFIJ*, *acfABC*, *tagADE*, and *ctxAB*), biofilm genes, glycerol uptake/utilization genes, and chitin utilization genes (24, 25).

A heatmap of the transcription patterns of flagellar genes in wild type, *flrBL36F*, and *flrBH135N* is shown (Fig. 3B). The heatmap depicts relative expression of each gene across the three strains compared to each other; the actual values are given in File S1. Transcription patterns of class III flagellar genes are even more apparent in this comparison, demonstrating upregulation in *flrBL36F* and downregulation in *flrBH135N*. These results are consistent with the L36F mutation causing increased FlrC-dependent transcription and with phospho-H135 being required for class III gene transcription.

Venn diagrams summarizing overlap between Δ*flaCEDB flrBL36F*-upregulated genes and *flrBL36F*-upregulated genes (25 genes), as well as between *flrBL36F*-upregulated genes and *flrBH135N*-downregulated genes (32 genes), give a clear picture of the FlrBC regulon in *Vc*. In addition to flagellar gene clusters *flgOP*, *flgT*, *flgBCDE*, *flgFGH*, *flgIJ*, *flgKL*, *flaA*, *fliKLMNOPQRflhB*, *motY*, and VC1384, it also includes VC1124, VC1125, VC1009, VC1064, VC1699, VCA0139, *sipA*, VCA0734, and VC0611-0619. We have constructed Δ*VC1125 Vc*, which had no apparent defect in motility (Fig. S3).

### FlrB is an asymmetric dimer with a unique PAS domain

**Fig 4:**
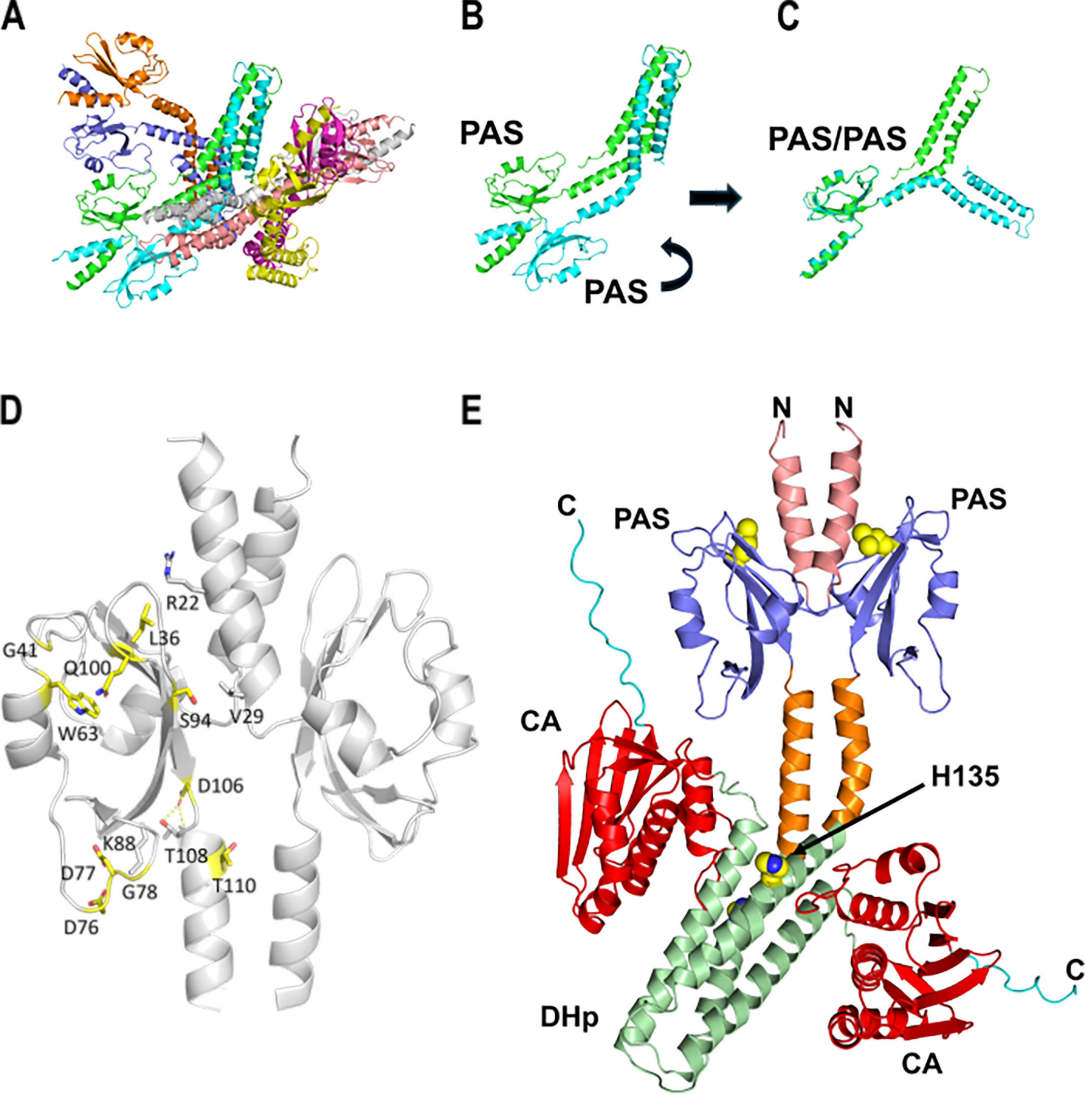
Structure of *Vc* FlrB. (**A**) The crystal structure of FlrB to 2.75 Å resolution. The asymmetric unit consists of eight chains/four dimers. (**B**) A single dimer reveals that FlrB was truncated after aa 192 and exists as an asymmetric dimer. (**C**) Superimposing the PAS domains within each monomer from panel **B** makes the asymmetric nature of the dimer more apparent. (**D**) The 10 residues within the PAS domain that were mutated and identified in this study are highlighted in yellow. Also shown are R22, V29, K88, and T108, which are predicted to interact with these residues. (**E**) Hybrid structure of full-length FlrB dimer, constructed from crystal structure (aa 1–192) and AlphaFold prediction (aa 193–351). PAS domain (purple), DHp domain (green), and CA domain (red), along with residue H135 indicated.

The crystal structure of FlrB was solved to 2.75 Å resolution; the asymmetric unit consisted of eight chains/four dimers (Fig. 4A; Table S1; PDB code 9p6i). Interestingly, although full-length FlrB was subjected to crystallization, crystals contained only FlrB aa 12–192 in the longest fragment (chain E). Three residues of the expression tag (Ser-Asn-Ala) and the first 11 N-terminal residues were disordered, and the C-terminus, including the catalytic domain CA (aa 193–351), was missing, likely due to peptide hydrolysis during crystallization. In the crystals, the FlrB^12-192^ fragments form an asymmetric dimer in which the two chains adopt different conformations (Fig. 4B); this can be seen if the PAS domains in each chain are superimposed (Fig. 4C). The extended helical DHp region forms two alternative kinks in the alpha helix at residue 130 in chain A and residue 125 in chain B, causing the two DHp regions to extend in opposite directions in this superimposition. This asymmetric fold has not been observed in dimer structures of other histidine kinases (HKs). The structure of the PAS domain is in good agreement (r.m.s.d. = 0.759 Å) with a further truncated FlrB PAS domain structure (aa 8–123) that was previously reported (27) (Fig. S4A and B).

FlrB PAS is a member of cluster 125 of identified PAS clusters across all domains of life (28). Members of this cluster have substituted Fα and Gβ elements of the prototypical PAS structure with a unique short loop region (Fig. S4C). Since Fα and Gβ are involved in ligand binding in other PAS clusters with known cofactors (e.g., heme), it is not clear how a cofactor might bind to FlrB PAS. Mapping PAS mutations that alter the activity of FlrB (Fig. 4D), we predict that some of these substitutions likely alter the local structure of FlrB. For example, L36 is in close proximity to R22 on the opposite monomer; the substitution of phenylalanine (L36F) may lead to a cation-π interaction that causes conformational changes between PAS and the N-terminus of the opposite monomer (Fig. S4D). D76 and D77 interact with K88; substitution of tyrosine residues (D76Y and D77Y) likely disrupts these interactions. D106 interacts with T108, and S94 interacts with V29 on the opposite monomer; substitutions (D106G and S94F) may disrupt these interactions. W63 and Q100 interact with each other, so substitutions W63L and Q100R likely disrupt this interaction.

We created a hybrid dimer structure by fusing the solved crystal structure to the AlphaFold prediction (29) for aa 193–351 (Fig. 4E). Because of the kink in DHp, the CA domain of one monomer is predicted to be in close proximity to the PAS domain, whereas on the other monomer, the kink in DHp positions the CA domain far from PAS. One possible scenario for PAS control over autophosphorylation could be sequestration of the CA domain by physical interaction with the PAS domain to prevent CA interaction with H135.

### The MS ring, but not heme, modulates FlrB activity

**Fig 5:**
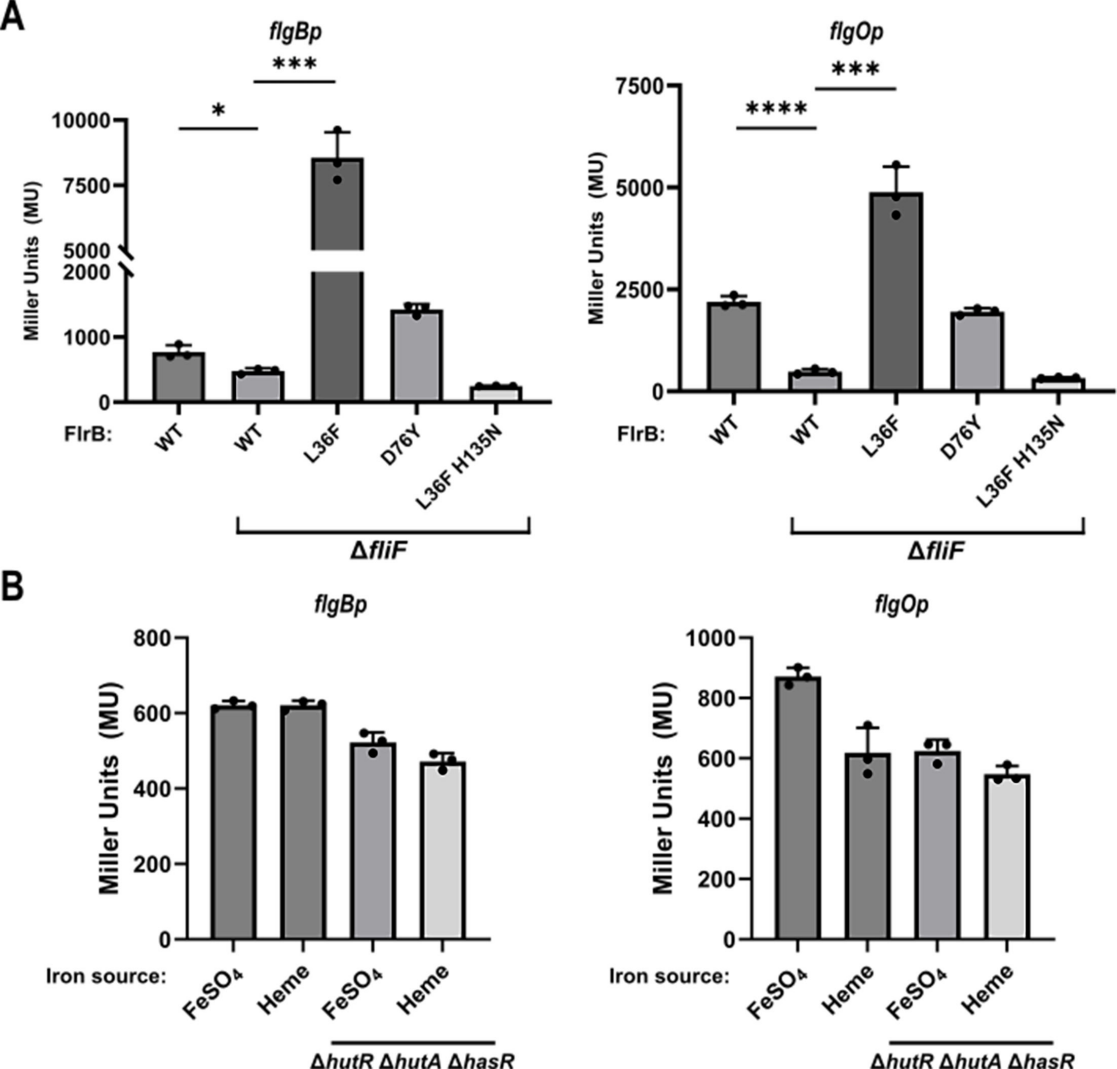
FlrB-dependent class III gene transcription responds to the presence of the MS ring, but not heme. (**A**) *Vc* strains KKV598 (wild type), KKV1247 (Δ*fliF*), KKV4107 (Δ*fliF flrBL36F*), KKV4105 (Δ*fliF flrBD76Y*), and KKV4106 (Δ*fliF flrBL36F H135N*), carrying pKEK332 (*flgB*p*-lacZ*, left) or pKEK782 (*flgO*p-*lacZ*, right) were assayed for β-galactosidase activity (see Materials and Methods) (unpaired, two-tailed Student’s *t*-test: *P ≤ 0.05; ***P ≤ 0.001; ****P ≤ 0.0001). (**B**) *Vc* strains KKV598 (wild type) and KKV4059 (Δ*hutA* Δ*hutR* Δ*hasR*), carrying pKEK332 (*flgB*p*-lacZ*, left) or pKEK782 (*flgO*p-*lacZ*, right), were grown with added FeSO4 or heme and assayed for β-galactosidase activity (see Materials and Methods).

Flagellar synthesis is initiated by the insertion of the MS ring into the cytoplasmic membrane and assembly of the export apparatus. Mutations in the MS ring (*fliF*) and export apparatus (*flhA*, *flhB*, and *fliPQR*) cause a decrease in FlrB/C-dependent transcription (18), implicating the assembly of the MS ring/T3SS as controlling FlrB activity. Measuring transcription from Class III promoters *flgB*p-*lacZ* and *flgO*p-*lacZ* in a Δ*fliF* strain, we found that transcription is significantly reduced in Δ*fliF* compared to wild type, confirming that the presence of MS ring stimulates FlrB-dependent transcription (Fig. 5A). The introduction of L36F into FlrB causes an increase in *flgB* and *flgO* transcription in the absence of FliF. Increased transcription is dependent on FlrB phosphorylation because transcription is eliminated in *flrB L36F H135N*. Finally, *flgB* and *flgO* transcription in Δ*fliF* is also increased with another PAS mutation (*D76Y*). These data are consistent with PAS mutations bypassing the requirement for the MS ring to increase FlrB-dependent transcription (i.e., under non-inducing conditions).

A report (27) identified heme as a ligand for FlrB and suggested that heme was a signal that activates FlrB-dependent transcription. If true, then blocking *Vc* heme import would diminish FlrB-dependent transcription. *Vc* heme import is dependent on two high-affinity heme transporters, HutA and HutR, and a third low-affinity transporter, HasR (30). We created *Vc* strains lacking either two (*hutA hutR*) or all three heme receptors (*hutA hutR hasR*). The double and triple mutant strains grew like wild type using FeSO_4_ as a sole iron source but were defective in growth with heme as a sole iron source (Fig. S5), indicating a lack of heme import. We measured transcription of *flgB*p-*lacZ* and *flgO*p-*lacZ* in the *hutA hutR hasR* strain in the absence or presence of 80 µM heme. There was little difference in *flgB*p and *flgO*p transcription, either in the wild-type or triple mutant strain, whether or not heme could be imported into *Vc* (Fig. 5B). These results demonstrate that extracellular heme does not modulate FlrB activity. The *hutA hutR hasR* mutant also showed similar swimming behavior to wild type in various concentrations of heme (Fig. S6). We conclude that the presence of the MS ring, but not heme, is important for FlrB-dependent transcription.

### FlrB localizes to the pole in a FlhF-dependent manner

**Fig 6:**
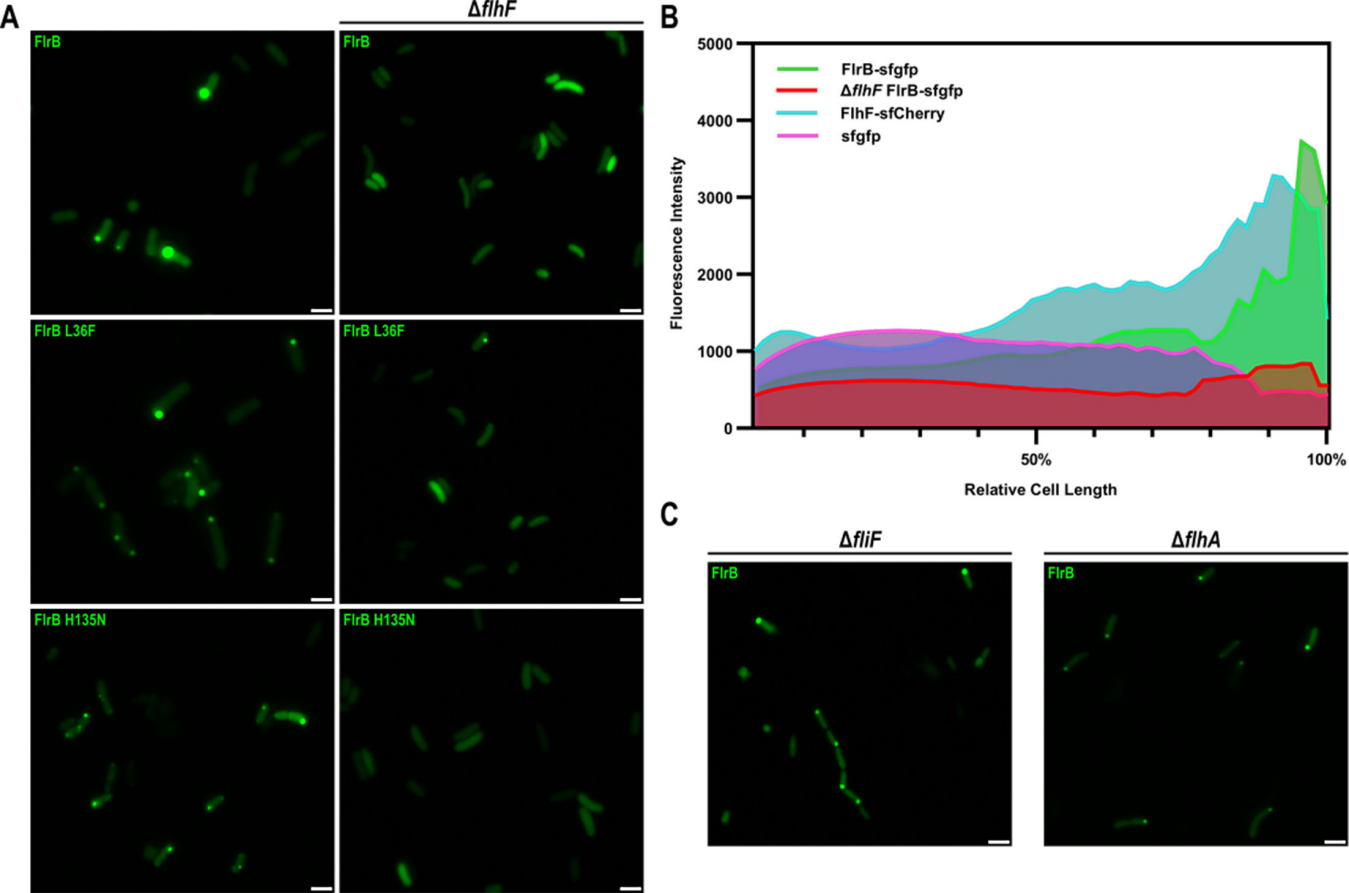
FlrB localizes to the cell pole in an FlhF-dependent manner. (**A**) *Vc* strains KKV176 (Δ*flrB*) or KKV4335 (Δ*flrB* Δ*flhF*) cells containing plasmids pKEK3494 (FlrB-sfgfp), pKEK3509 (FlrB L36F-sfgfp), or pKEK3510 (FlrB H135N-sfgfp) were grown in the presence of 0.2% arabinose and observed by fluorescence microscopy. Scale bars = 1 µm. (**B**) *Vc* strains KKV176 (Δ*flrB*) and KKV4335 (Δ*flrB* Δ*flhF*) containing plasmid pKEK3494 (FlrB-sfgfp), KKV1560 (Δ*flhF*) containing plasmid pKEK3590 (FlhF-sfcherry), or KKV176 (Δ*flrB*) containing plasmid pKEK3093 (sfgfp) were grown in the presence of 0.2% arabinose and imaged by fluorescence microscopy. Cells (*n* = 100) were scanned for fluorescence intensity along the major axis using the line scan mode of ImageJ analysis software (ImageJ version 1.37). (**C**) *Vc* strains KKV3412 (Δ*fliF* Δ*flrB*) and KKV3432 (Δ*flhA* Δ*flrB*) containing plasmid pKEK3494 (FlrB-sfgfp) were grown in the presence of 0.2% arabinose and imaged by fluorescence microscopy. Scale bars = 1 µm.

Since the presence of the MS ring stimulates FlrB-dependent transcription, we wondered whether FlrB localizes to the cell pole to monitor MS ring assembly. We expressed FlrB-sfgfp in Δ*flrB Vc* and visualized localization via fluorescence microscopy. FlrB localizes to the cell pole in the majority of cells (Fig. 6A), which was quantitated by measuring fluorescence intensity over cell length, in comparison to cytoplasmic sfgfp alone (Fig. 6B). Interestingly, “on” (L36F) and “off” (H135N) forms of FlrB-sfgfp also localize to the cell pole (Fig. 6A), suggesting that FlrB activation state does not influence polar localization.

**Fig 7:**
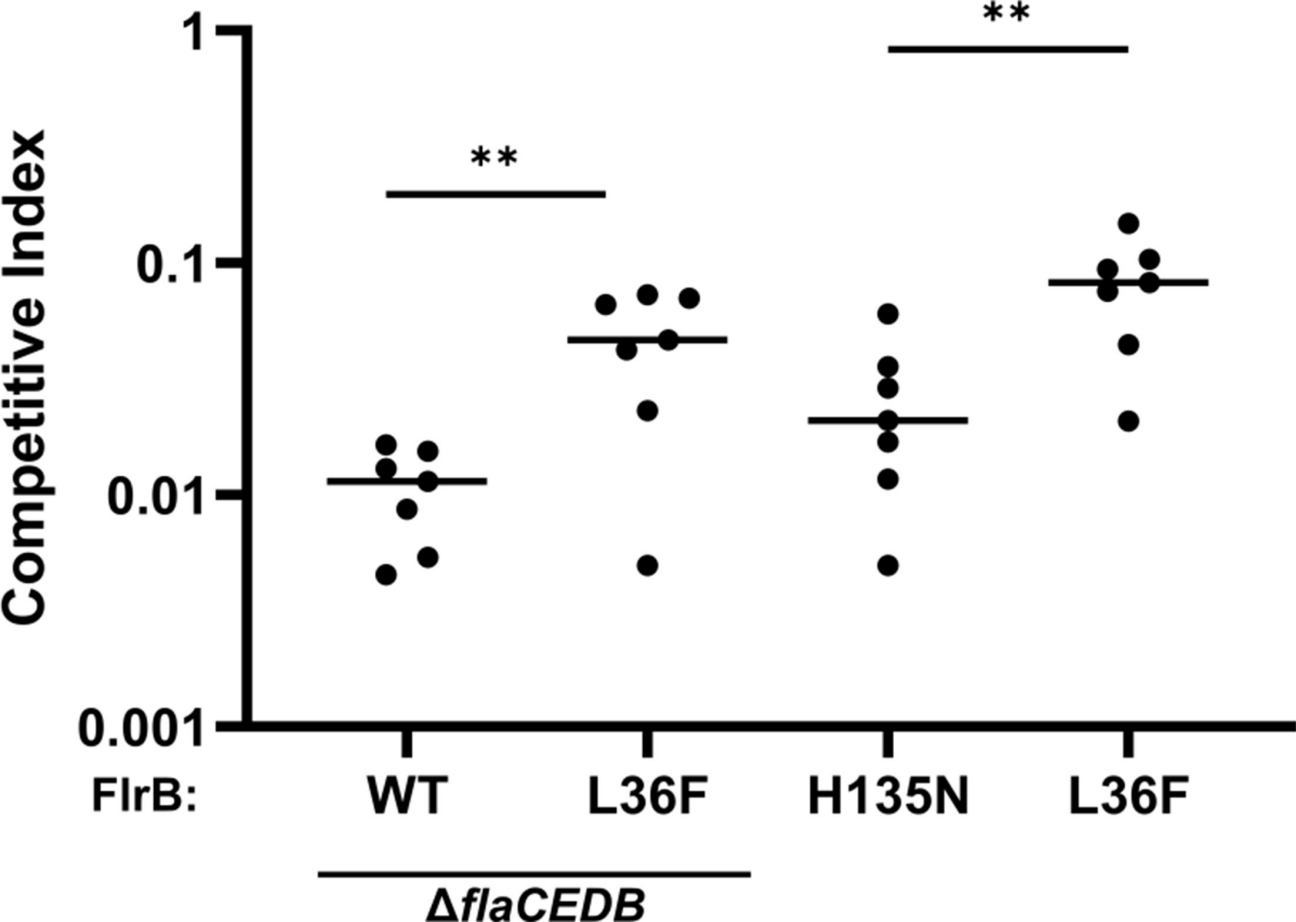
“On” or “off” forms of FlrB inhibit *Vc* intestinal colonization. *Vc* strains KKV2530 (Δ*flaCEDB*), KKV2579 (Δ*flaCEDB flrBL36F*), KKV3415 (*flrBH135N*), and KKV3288 (*flrBL36F*) were coinoculated with the wild-type strain O395 perorally into infant mice. Intestinal homogenates were recovered at 22 h post-inoculation, and CFU of wild-type and mutant strains was determined. The CI is given as the output ratio of mutant:wild type divided by the input ratio of mutant:wild type; each value shown is from an individual mouse. (unpaired, two-tailed Student’s *t*-test: **P ≤ 0.01).

The MS ring is not required for FlrB polar localization, since FlrB-sfgfp localizes to the cell pole in Δ*fliF Vc* (Fig. 6C). Likewise, an export apparatus mutation (*flhA*), which is required for FlrB activation (18), does not prevent FlrB-sfgfp polar localization (Fig. 6C). Mutations in additional flagellar components (*flgG* and *flgI*) do not affect FlrB-sfgfp polar localization (Fig. S7). FlhF is known to be required for polar flagellar localization in *Vibrio* spp. (31, 32), and it also localizes to the cell pole (Fig. 6B and 7). Interestingly, FlrB-sfgfp failed to localize to the pole in Δ*flhF Vc* (Fig. 6A). This was quantitated by measuring fluorescence intensity over cell length in Δ*flhF* cells (Fig. 6B). FlrB-sfgfp in Δ*flhF Vc* was diffusely localized throughout the cell, similar to sfgfp alone. “On” (L36F) as well as “off” (H135N) forms of FlrB also failed to localize to the pole in Δ*flhF Vc* (Fig. 6A). The diffuse signal in Δ*flhF* demonstrates that polar localization in a wild-type background is not due to overexpression of FlrB-sfgfp. These results demonstrate that FlrB polar localization is dependent upon the polar targeting protein FlhF.

### “Off” or “on” forms of FlrB inhibit intestinal colonization

Our cumulative data indicate that PAS mutations increase phospho-FlrB levels, which increase phospho-FlrC-dependent class III transcription. We also demonstrated that FlrB H135N cannot be phosphorylated and fails to stimulate class III transcription. To determine if FlrB in “off” (H135N) or “on” (L36F) forms affects *Vc* virulence, we utilized an infant mouse competition assay. Colonization of the infant mouse intestine is correlated with *Vc*’s ability to cause human disease (33).

The non-motile Δ*flaCEDB* strain exhibits an approximate 100-fold defect in intestinal colonization (Fig. 7; competitive index [CI] = 0.0107). The addition of *flrBL36F* into this genetic background allows *Vc* to regain motility (Δ*flaCEDB flrBL36F*) and increase intestinal colonization, although it is still approximately 20-fold defective for intestinal colonization (CI = 0.0466).

The *flrBH135N* (“off”) and *flrBL36F* (“on”) mutations in an otherwise wild-type background strain were also measured for intestinal colonization. *flrBH135N* showed a 40-fold defect (CI = 0.0256). Interestingly, *flrBL36F* was also approximately 10-fold defective for intestinal colonization (CI = 0.0810). These experiments illustrate enhanced intestinal colonization by motile strains. Additionally, the colonization defect of both “off” and “on” *flrB* strains demonstrates that FlrB in either the inactive or hyperactive form is deleterious for *Vc* virulence.

## DISCUSSION

Flagellar-mediated motility contributes to the ability of *Vibrio* spp. to obtain nutrients through chemotaxis, to colonize surfaces, and to colonize hosts to cause disease (1, 34). The *Vc* transcription hierarchy ensures that flagellar genes are expressed in a step-wise fashion. The *Vc* two-component FlrBC system controls class III gene transcription, which encodes flagellar components in the rod-ring-hook structure of the basal body (14, 15, 23). FlrBC-dependent transcription thus lies embedded within the hierarchy, with activation of class III transcription dependent on previous class II gene expression and assembly (18). Additionally, cessation of class III transcription likely occurs during class IV expression to prevent accumulation of excess flagellar components. *Vc*’s ability to turn on and off class III transcription is mediated through the histidine kinase FlrB.

In this study, we showed that FlrB autophosphorylates at H135 in the DHp domain. We previously demonstrated that FlrB-P transfers phosphate onto D54 in the FlrC amino-terminus, which facilitates σ^54^-dependent transcription at class III promoters (12). FlrB is a cytoplasmic protein and therefore likely controlled by a cytoplasmically localized signal. It has been proposed that FlrB recognizes an assembled secretion apparatus of MS ring-rotor-T3SS (18), and we confirmed that the presence of the MS ring stimulates FlrB-dependent transcription.

Given that the flagellum is located at the cell pole, we reasoned that FlrB would also be polarly localized to monitor assembly. We demonstrate that FlrB localizes to the cell pole, and polar localization depends on FlhF (Fig. 6 and 7). FlhF (16, 35) recruits the earliest flagellar component, FliF (MS ring), to the cell pole (32). FlrB localized to the pole even in bacteria lacking FliF or the flagellar T3SS (FlhA), but FlrB was diffusely distributed throughout the cell in the absence of FlhF. We previously showed downregulation of class III genes in a Δ*flhF* strain (16), which is consistent with FlrB polar localization being required for class III transcription activation. Interestingly, both “on” (L36F) and “off” (H135N) forms of FlrB localize to the pole, suggesting that polar localization is independent of phosphorylation, but this requires further investigation.

Another cytoplasmic histidine kinase, the chemotaxis protein CheA, is recruited to the *Vc* cell pole by ParP (36), which is itself recruited to the pole by ParC (37). ParC is a member of the ATP-binding “ParA family” proteins, similar to FlhG. FlhG localizes to the cell pole and also interacts with FlhF to modulate its presence at the cell pole (31, 37). Both ParC and FlhG, along with a third ATP-binding protein, ParA1, are anchored to the *Vc* cell pole by HubP (38). HubP also interacts with FlhF, although interaction is not required for FlhF to localize to the cell pole (31). FlhF recruits the MS ring (FliF) to the cell pole, which ensures polar localization of the flagellum (32). We have shown here that FlhF is required for FlrB polar localization, but FliF is not required. However, FliF is required for FlrB-dependent class III transcription. The requirements for polar localization of FlrB and how this affects activity deserve further investigation.

**Fig 8:**
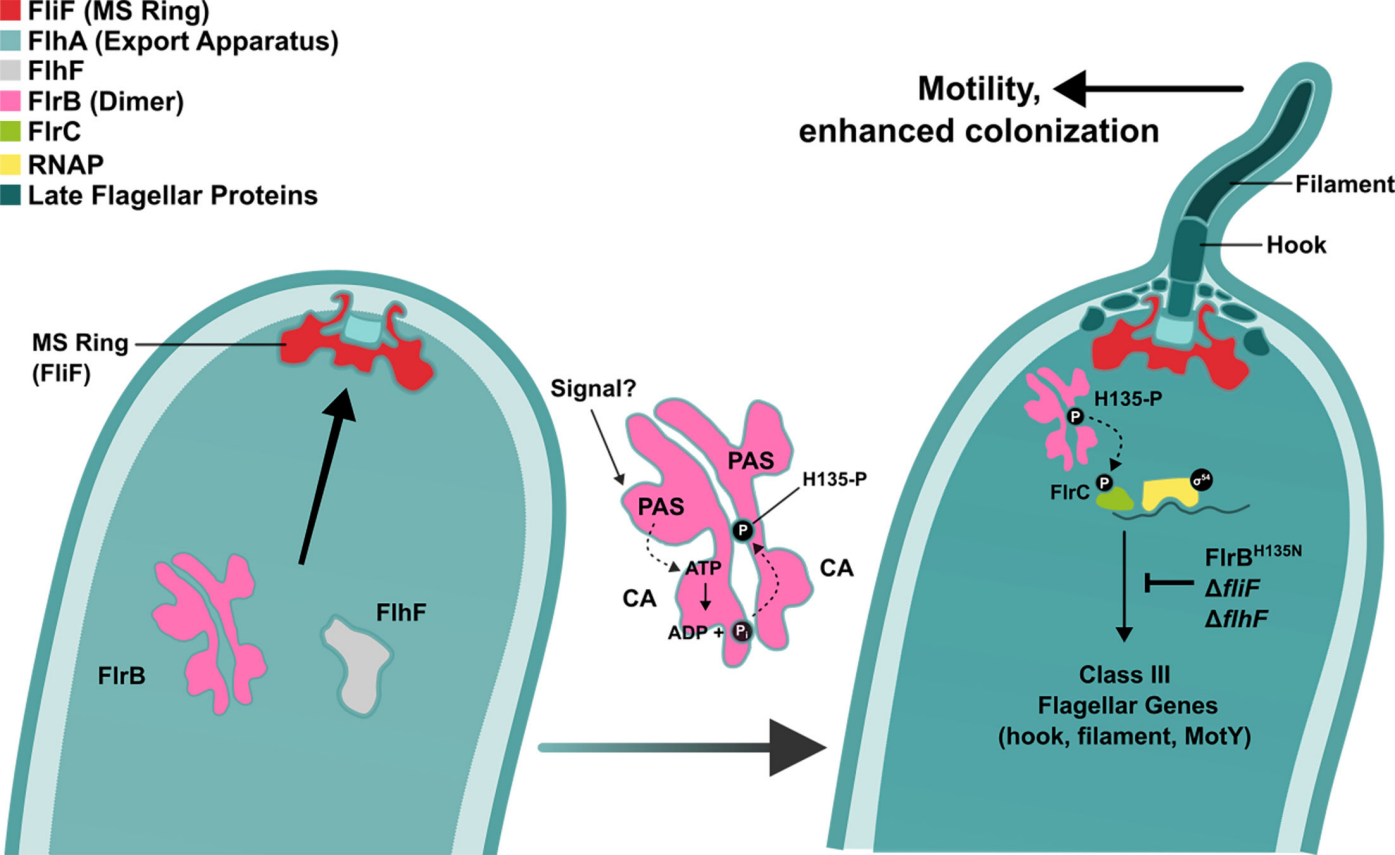
Model of FlrB-dependent regulation of class III flagellar gene transcription in *Vc*. Polar localization of FlrB depends on FlhF. The PAS domain controls autophosphorylation of FlrB, which is governed by an (unknown) external signal, related to the presence of an early flagellar structure. FlrB autophosphorylates at the conserved histidine residue H135. Phospho-FlrB transfers the phosphate to the response regulator FlrC, which activates σ^54^-dependent (class III) flagellar gene promoters. Class III genes encode the rod-hook structure and the core flagellin FlaA, which are essential for *Vc* motility and enhanced intestinal colonization. It is unknown whether FlrB undergoes *cis*- or *trans*-autophosphorylation (39, 40).

Presumably, FlrB polar localization is necessary to monitor flagellar assembly, which leads to FlrB phosphorylation, but the signal that stimulates FlrB autophosphorylation and phosphotransfer to FlrC is unclear (Fig. 8). A recent report suggested that external heme stimulates FlrB autophosphorylation (27), but our results do not support this hypothesis. Inactivation of all three *Vc* heme receptors prevented heme uptake, yet FlrB-dependent transcription remained similar to a wild-type strain (Fig. 5B), and motility was unaffected (Fig. S6). Additionally, the FlrB PAS domain lacks Fα and Gβ regions (Fig. S4C), which are critical for heme binding in other PAS domains (Fig. S4E), so it is unclear how FlrB would bind heme.

The results shown here support the involvement of the PAS domain in regulating FlrB autophosphorylation and class III gene activation. We identified 10 PAS domain mutations (Fig. 4D) that facilitated the motility of Δ*flaCEDB Vc*. These mutations increase class III transcription, both in Δ*flaCEDB* and wild-type backgrounds. The PAS mutations stimulate class III transcription even in Δ*fliF* (MS ring) *Vc*. These results are consistent with the PAS mutations representing “on” forms of FlrB that are active even under non-inducing conditions. As predicted from this hypothesis, FlrB L36F exhibits higher levels of autophosphorylation than wild-type FlrB *in vitro* (Fig. 2A).

H135 is C-terminal to the kink in the alpha helix in the FlrB^1-192^ crystal structure. In our hybrid model of full-length FlrB (Fig. 4E), the CA domain of one monomer lies in close proximity to H135 in the opposite monomer, as well as to the PAS domain of the same monomer. One scenario of control over autophosphorylation: interactions between PAS and CA domains within one monomer hold CA in an inactive state, and disruption of these interactions frees CA to catalyze phosphate transfer onto H135 on the opposite monomer. More structural studies with the PAS mutant and full-length FlrB are necessary to test this hypothesis.

The PAS mutations that stimulate FlrB-dependent transcription were isolated in Δ*flaCEDB Vc*, which is non-motile despite expressing the FlaA flagellin (7). Overexpression of FlaA in Δ*flaCEDB* restores motility (Fig. S2), indicating that the failure of Δ*flaCEDB* to swim is due to the lack of sufficient FlaA. However, transcription of class III genes, including *flaA*, is similar between Δ*flaCEDB* and wild type (Fig. 3A), suggesting that “wild-type” FlaA levels are insufficient for motility. The Δ*flaCEDB Vc* strain synthesizes a truncated filament (Fig. 1D), and it has recently been shown by cryo-electron microscopy (W. Guo, S. Zhang, J. H. Park, V. Stanton, M. Asp, H. Herrera, J.-S.B.Tai, J. Yue, J. Wang, S. Wu, J. Yan, K. E. Klose, F. Yildiz, and J. Liu, unpublished data) that FlaA forms a short segment at the base of the filament that acts as a bridge between the hook/filament FlgK/FlgL proteins and the other flagellins. We hypothesize that this short FlaA segment in the filament can only be extended by excess levels of FlaA. The *flaA* transcript has a 3′ UTR that contains the *flaX* sRNA (41, 42), which negatively regulates *flaA* translation. This mechanism may prevent sufficient FlaA levels to build an entire filament, which might be overcome by elevated FlrB-dependent transcription.

*Vc* intestinal colonization is critical for cholera pathogenesis, and the “on” (L36F) and “off” (H135N) forms of FlrB cause defects in *Vc*’s ability to colonize the intestine (Fig. 7). The *flrBH135N* strain is non-motile, which is known to lead to defects in colonization; in addition, *flrBH135N* transcribed reduced levels of virulence genes (*tcp* and *ctx*) *in vitro* (File S1), which could contribute to its poor colonization *in vivo*. The Δ*flaCEDB* strain is also non-motile and defective for colonization, and the introduction of the L36F mutation into this strain facilitated increased colonization, consistent with a role for motility in intestinal colonization. Still, the *Vc* strains with L36F PAS mutations were defective for colonization, demonstrating that both “on” and “off” forms of FlrB inhibit the virulence of *Vc*. These results are consistent with the dynamic process of intestinal colonization, where flagellar-based motility (“on”) facilitates arrival at the colonization site, but extended colonization favors the cessation of motility (“off”), and FlrB enables switching between both modes.

## MATERIALS AND METHODS

### Bacterial strains, plasmids, and growth conditions

A complete list of primers and plasmids used is provided in Table S2 and S3. Details on plasmid and strain construction are included in the supplemental material. All plasmid constructs were verified by sequencing. A complete list of *Vc* strains is provided in Table S4. All chromosomal deletions were confirmed by sequencing. Growth conditions for the various experiments are detailed in the supplemental material.

### Whole-genome sequencing

Total genomic DNA was extracted from *Vc* strains KKV2579 and KKV2580 and subjected to short-read (Illumina) sequencing. Genomes were annotated as described in the supplemental material.

### Western immunoblot and beta-galactosidase assays

Rabbit polyclonal antisera to *Vc* FlaA (8) were used in immunoblots (1:2,000). Membranes were developed with ECL-plus. *Vc* strains carrying the promoter-*lacZ* plasmids listed in Table S3 were grown at 37°C to mid-log (OD_600_ 0.4–0.8) and assayed for β-galactosidase activity by the method described by Miller (43). Each strain was measured in triplicate.

### RNA extraction, sequencing, and analysis

Strains were grown in triplicate in 5 mL of Luria broth (LB) at 37℃ in a roller drum until mid-log (OD_600_ 0.4–0.6) before RNA extraction using TRIZOL reagent. The purity of RNA was confirmed using a Nanodrop. Sequencing was performed at the Genome Sequencing Facility at UT Health-San Antonio. Raw reads were assessed for quality using FastQC. Details on RNAseq analyses can be found in the supplemental material.

### Autophosphorylation of FlrB

Wild-type, L36F, and H135N forms of FlrB were purified as described in the supplemental material. Autophosphorylation was detected using the bio-orthogonal ATP analog ATPγS to produce thiophosphorylated FlrB. The protein was then treated with the alkylating agent p-nitrobenzyl mesolate (PNBM), and the resulting PNBM-derivatized thiophosphate epitope was identified immunochemically, as described in detail in the supplemental material (20).

### X-ray crystal structure of FlrB

FlrB was purified and subjected to crystallization, and the crystal structure was solved as described in the supplemental material. The crystal structure statistics are given in Table S1 (PDB 9P6I).

### Mouse intestinal colonization assay

We utilized a competitive index assay for intestinal colonization in 5-day-old CD-1 suckling mice as described previously (44) and detailed in the supplemental material. The CI is the output ratio of mutant:wild type divided by the input ratio of mutant:wild type. Prism 5.0b was used for statistical analyses using Student’s paired *t*-test.

### Fluorescence microscopy

*Vc* strains expressing protein fusions to sfgfp/sfcherry were imaged with an Olympus BX43 microscope, and fluorescence intensity was quantitated via ImageJ (cells analyzed = 100 per strain). Details of imaging techniques are in the supplemental material.

## Data Availability

The sequence data sets have been deposited in GenBank at NCBI with accessions JBBMZZ000000000 and JBBMZY000000000. The RNA-seq data have been deposited in NCBI Gene Expression Omnibus (45) through GEO Series accession number GSE302736. The FlrB crystal structure coordinates and processed diffraction data have been deposited to rcsb.org Protein Data Bank under code 9P6I.

## SUPPLEMENTAL MATERIAL

File S1 mbio.02379-25-s0001.xlsx RNAseq data, including raw reads for conditions featured in Fig. 3.

Supplemental Material mbio.02379-25-s0002.pdf Supplemental text, figures, and tables.

ASM does not own the copyrights to Supplemental Material that may be linked to, or accessed through, an article. The authors have granted ASM a non-exclusive, world-wide license to publish the Supplemental Material files. Please contact the corresponding author directly for reuse.
