# Supplementary material for: The PAS domain of the polarly localized histidine kinase FlrB in Vibrio cholerae controls class III flagellar transcription and contributes to intestinal colonization
Source: mBio. 2025 Sep 22;16(11):e02379-25. doi: 10.1128/mbio.02379-25 (PMC12607845; doi:10.1128/mbio.02379-25)
Supplement: Supplemental Material — Supplemental text, figures, and tables. [file mbio.02379-25-s0002.pdf]

Supporting Information for

The PAS domain of the Polarly-Localized Histidine Kinase FlrB in *Vibrio cholerae* Controls Class III Flagellar Transcription and Contributes to Intestinal Colonization

Venus Stanton[1], Bradley Himes[1], Adrian Mejia-Santana[1], Mark Eppinger[1], Jesus Romo[1], Jiawei Xing[2], Igor B. Zhulin[2], Hong Cai[1], Yufeng Wang[1], Nicole I. Inniss[3], George Minasov[3], Karla J. F. Satchell[3], and Karl E. Klose[1#]

Karl E. Klose[1#]
Email:Karl.Klose@utsa.edu

This PDF file includes:

Supporting Text
Figures S1 through S7
Tables S1 through S4
SI References

Supporting Information

Plasmid construction:

flrB alleles were PCR amplified from Vc strains using primers flrBp F FC and flrB R FC, then cloned into pKEK2200 PCR amplified by universal 1 and universal 2 by In Vivo Assembly (1, 2) to form pKEK2158 (WT *flrB*), pKEK2164 (*flrB L36F*) and pKEK2442 (*flrB D76Y*). Site Directed mutagenesis was performed on pKEK2158, pKEK2164, and pKEK2442 with primers flrB H135N F and flrB H135N R to form pKEK2395 (*flrB H135N*), pKEK2405 (*flrB L36F H135N*), and pKEK2461 (*flrB D76Y H135N*).

The deletions in *flaC*, *flhA, and VC1124* were constructed by Splicing by Overlap Extension (3) with the primers listed in Table S2, and the resulting deletion fragments were either inserted into pKEK2202 or pKEK2094 PCR-amplified with universal primers by IVA (2), resulting in pKEK1994 (Δ*flaC*) and pKEK2377 (Δ*flhA*), or cloned by Golden Gate assembly into pKEK2949 (*BsaI*) resulting in pKEK3642 (Δ*VC1125*). pKEK1994 and pKEK1854 were PCR amplified with DelflaC+AbR R and DelflaC+AbR F, and DelflaEDB+AbR R and DelflaEDB+AbR F, respectively, and FRT-SpcR-FRT fragment was PCR-amplified from SAD033 with primers ABD123 and ABD124 (4), and inserted into pKEK1994 and pKEK1854 by IVA (2) to make pKEK2286 (Δ*flaC*::FRT-SpcR-FRT) and pKEK2227 (Δ*flaEDB*::FRT-SpcR-FRT). pKEK2377 was PCR amplified with DelflhA R AbR and DelflhA F AbR, and FRT-KanR-FRT fragment was PCR-amplified from SAD034 with primers ABD123 and ABD124 (4), and inserted into pKEK2377 by IVA (2) to make pKEK2402 (Δ*flhA*::FRT-KanR-FRT). The Δ*ctxA* deletion was assembled by NEBuilder HiFi into pKEK2200 with fragments generated from primers listed in Table S2, resulting in pKEK2154 (Δ*ctxA*). Δ*flgG* and Δ*flgI* were synthesized as gblocks (IDT) in which the entire coding sequences were removed; these were cloned into pKEK229 resulting in pKEK2013 (Δ*flgG*), pKEK2014 (Δ*flgI*),

The FlrB-sfgfp expression plasmid was made in several steps. First *flrB*, *flrB H135N*, and *flrB L36F* were cloned into pKEK3093 by PCR amplification with the primers listed in Table S2 of two fragments that removed an internal *Bsa*I site and allowed for Golden Gate assembly, resulting in pKEK3490 (pBAD-*flrB*), pKEK3504 (pBAD-*flrB H135N*) and pKEK3503 (pBAD-*flrB L36F*). sfgfp was PCR amplified from pFY5790 with primers sfgfp F NcoI SphI GG BsaI + sfgfp R SalI GG BsaI and cloned into pKEK3093 by Golden Gate Assembly resulting in pKEK3370 (pBAD-sfgfp). Finally, pKEK3490, pKEK3503, and pKEK3504 were digested with *Sph*I and *Sal*I, and ligated to the *Sph*I-*Sal*I fragment from pKEK3370 to make pKEK3493 (pBAD-*flrB*-sfgfp), pKEK3509 (pBAD-*flrB L36F*-sfgfp) and pKEK3510 (pBAD-*flrB H135N*-sfgfp). The FlhF-sfcherry expression plasmid was made by first PCR amplification of *flhF* with primers VCflhF NcoI F BsaI GG and VcflhF R SalISphI BsaI and cloned into pKEK3093 by Golden Gate Assembly resulting in pKEK3607 (pBAD-*flhF*). sfcherry from pKEK3398 (5) was PCR amplified with sfcherry F NcoI SphI GG BsaI and sfcherry R SalI GG BsaI and cloned into pKEK3093 by Golden Gate Assembly resulting in pKEK3524 (pBAD-sfcherry). Finally pKEK3607 was digested with *Sph*I and *Sal*I, and ligated to the *Sph*I-*Sal*I fragment from pKEK3524 to make pKEK3590 (pBAD-*flhF*-sfcherry).

V. cholerae strain isolation and construction:

The *Vc* spontaneous motile mutants were isolated by extended incubation of KKV2530 or KKV3159 (Δ*flaCEDB*) in motility agar, and streak purification of motile variants. The *flrB* mutations were introduced back into the *Vc* genome by conjugation into Δ*flrB Vc* strains, selection for integration, and then counter-selection on sucrose-containing media for loss of plasmid. Most *Vc* mutants in the classical O1 O395 strain were generated through allelic exchange using the backbone plasmids pKEK229 (6), pKEK2200 (1), pKEK2202 (1), or pKEK2949 via *sacB* counterselection. The A1552 Δ*ctxA* strain KKV3000 was generated in the same manner. Vc mutants in KKV3000 background were generated through natural transformation/MUGENT (4) with PCR product. The Δ*flaEDB*::FRT-SpcR-FRT mutant KKV3077 was generated in this manner, then the SpcR gene was removed by pBAD-FLP (7) to make strain KKV3104. Δ*flaC*::FRT-SpcR-FRT was subsequently moved into this strain by the same method to create KKV3153, then the SpcR gene was removed by pBAD-FLP (7) to make strain KKV3159. The heme transport mutant KKV4059 was constructed by natural transformation, using the technique outlined in (8) and gDNA from ARM313 and ARM315, followed by allelic exchange with pAMS4.

Growth conditions:

Luria-Bertani (LB) media was used for both agar plates and liquid cultures. Carbenicillin at 200 μg/mL, Streptomycin at 100 μg/mL, Kanamycin at 50 μg/mL, chloramphenicol at 2 mg/ml were used as selective antibiotics when necessary. Agar plates consisting of LB with 0.3% agar incubated at 30°C were used to measure motility. For electron microscopy, *V. cholerae* strains were inoculated into M9 Minimal Media supplemented with Iron Sulfate, Sodium Citrate, and Sodium Pyruvate as described (9). For growth on various iron sources, media was first depleted of iron by addition of 100 μg/ml ethylenediamine-di-(o-hydroxyphenylacetic acid) (EDDA), then supplemented with 40 μM ferrous sulfate or 5 mM hemin.

Whole genome sequencing:

Total genomic DNA was extracted from *V. cholerae* strains KKV2579 and KKV2580 using the QIAamp DNA Mini Kit (Qiagen) according to the manufacturer's instructions. gDNA was subjected to short-read (Illumina) sequencing. Paired-end libraries were prepared with the NxSeq AmpFREE Low DNA Library Kit (Lucigen) with a 250-bp read length and sequenced using the MiSeq Reagent kit (v2) (500-cycle). Sequencing reads in the fastq format were imported into Galaxy (10), and default software parameters were used for all analysis. FastQC (http://www.bioinformatics.babraham.ac.uk/projects/fastqc) was used to determine read quality. Sequencing reads were assembled using SPAdes (11), and the resulting contigs were evaluated with QUAST (12). Contigs smaller than 1,000 bp were filtered out and the remaining 77 and 75 contigs for KKV2579 and KKV2580, respectively, were annotated using the NCBI Prokaryotic Genome Annotation Pipeline (13).

RNAseq Analyses:

The analyses of RNA-Seq data were carried out using CLC Genomics Workbench 24 (Qiagen). Low-quality sequences and adapters were trimmed. Trimmed reads were mapped to the *V. cholerae O395* genome assembly ASM964613v1. The reads were assigned to the transcripts using the EM algorithm. The TMM normalization (14) in EdgeR (15, 16), and multi-factorial statistics based on a negative binomial Generalized

Linear Model were carried out for differential expression analysis. The Benjamini-Hochberg procedure (17) was used to control the false discovery rate (FDR). Genes with FDR-adjusted p-value <0.05 and log2 fold changes of more than 1 or less than -1 were considered to be differentially expressed. KEGG (18) pathway enrichment analysis was performed using the KOBAS program (19). The Benjamini and Hochberg procedure was used for multiple-testing correction for pathway analyses, with a FDR < 0.05 cutoff criterion.

Purification of FlrB:

The wildtype *flrB* gene (NCBI Reference Sequence: WP_001881834.1), *flrBH135N*, and *flrBL36F* were synthesized in *pET28b* with an N-terminal His tag (GenScript). *E. coli* BL21 (DE3) containing the *flrB* expression plasmids was grown in TB broth supplemented with 0.4% glycerol (v/v) containing 50 µg/mL kanamycin at 37°C with shaking (250 rpm). Protein expression was induced by 200 µM isopropyl β-D-thiogalactopyranoside (IPTG) at OD_{600} 1.0. The culture was lowered to 25˚C and cells were harvested by centrifugation (4 230 x g, 10 min, 4 °C) after ~20 hours. The pellet was resuspended in 50 mM Tris pH 8.0, 500 mM sodium chloride, and 20% (v/v) glycerol, then flash frozen at -80˚C. While thawing, lysozyme was added and the cells were disrupted with three freeze thaw cycles. Deoxyribonuclease I and 10 mM magnesium chloride were added, cells were incubated for 2 hours on ice, and cellular debris was removed by centrifugation (20 000 x g, 30 min, 4˚C). The supernatant was injected onto a nickel charged Sepharose fast-flow column (Cytiva) pre-equilibrated in 50 mM Tris pH 8.0, 500 mM sodium chloride, 50 mM imidazole, and 20% (v/v) glycerol. The protein eluted as a step gradient with buffer supplemented with 300 mM imidazole. The fractions containing FlrB were pooled and subjected to a Superdex 200 size-exclusion column (Cytiva) equilibrated in 50 mM Tris pH 8.0, 150 mM potassium chloride, and 20% (v/v) glycerol. The FlrB fractions were collected and concentrated to 12 mg/ml by Bradford analysis. FlrB was obtained at 33 mg per L of cell growth. The protein was flash cooled and stored at -80˚C.

FlrB x-ray structure determination.

The full-length open reading frame for *flrB* (*VC2136*) was amplified from genomic DNA of *Vc* strain O1 biovar El Tor str. N16961 (NCBI reference sequence NC_002505.1) and the PCR product was cloned using ligation independent cloning as described previously into pMSCG53 vector to expressed recombinant protein with an N-terminal 6×His tag followed by a tobacco etch virus (TEV) protease cleavage site (20). Recombinant FlrB was expressed in *Escherichia coli* BL21(DE3)-Magic cell in 3 liters High Yield M9 SeMet media (Medicilon Inc.) supplemented with 200 µg/ml ampicillin and 50 µg/ml kanamycin at 25°C. The bacterial pellets collected by centrifugation were resuspended in 50 mM Tris pH 8.3, 0.5 M NaCl, 10% glycerol, 0.1% IGEPAL CA-630, flash frozen, and stored at −30°C until purification. Thawed cells were lysed by sonication and lysate was clarified by centrifugation. The protein was purified using an ÅKTAxpress system (GE Healthcare) as previously described with some modifications (21). The supernatant was loaded three times onto a HisTrapFF (GE Healthcare) column in loading buffer (10 mM Tris-HCl pH 8.3, 500 mM NaCl, 1 mM Tris (2-carboxyethyl) phosphine (TCEP), and 5% glycerol). The column was washed with 10 column volumes (cv) of loading buffer and 10 cv of washing buffer (10 mM Tris-HCl pH 8.3, 1M NaCl, 25 mM imidazole, 5% glycerol). The protein was eluted with elution buffer (10 mM Tris pH 8.3, 500 mM NaCl, 1 M imidazole) and then loaded onto a Superdex 200 26/600 column and separated in loading buffer. The 6xHis-tag was cleaved by recombinant TEV

protease in ratio 1:20 (protease:protein) overnight at 22°C and passed over a HisTrapFF column using loading buffer with 5 mM imidazole. The cleaved protein was dialyzed into 10 mM Tris-HCl pH 8.3, 1 M NaCl and concentrated to 6.5 mg/ml. Some precipitated protein was removed by filtration and protein was set up for crystallization in 2-µl crystallization drops (1 µl protein: 1 µl reservoir solution) in 96-well crystallization plates (Corning) using commercially available screens. Diffraction-quality crystals were obtained from 0.37 M Sodium sulfate, 0.1 M MES pH 6.5. (condition G8 of the Anions Suite from Molecular Dynamics). The crystal was cryoprotected in 2 M Lithium sulfate and flash frozen in liquid nitrogen for data collection.

Diffraction data were collected on the 21-ID-D beamline of the Life Science Collaborative Access Team (LS-CAT) at the Advanced Photon Source, Argonne National Laboratory, USA. The data set was processed and scaled using the *HKL*-3000 suite (22). The structure was solved by the single-wavelength anomalous dispersion (SAD) method using selenomethionine-derivatized protein. The initial solution went through several rounds of refinement in *REFMAC* version 5.7.0032 (23) and manual model correction using *Coot* (24). The water molecules were generated using *ARP*/*wARP* (25) and ligands were added to the model manually during visual inspection in *Coot*. Translation–libration–screw (TLS) groups were created by the *TLSMD* server (https://skuld.bmsc.washington.edu/~tlsmd/; (26) and TLS corrections were applied during the final stages of refinement. *MolProbity*(https://molprobity.biochem.duke.edu/; (27) was used to monitor the quality of the model during refinement and for final validation of the structure. Structure analysis and figure preparation was performed with PyMol.

Autophosphorylation of FlrB:

Autophosphorylation was detected using the ATP analog ATPγS to produce thiophosphorylated FlrB. (28). 1 mM ATPγS was added to 15 µM FlrB in a 15 µL volume in 50 mM Tris pH 7.4, 50 mM KCl, 10 mM $MgCl_2$. After incubation at RT, reaction was quenched with 3 µL of 500 mM EDTA, pH 8.0. Then 1 µL of 50 mM PNBM in 100% DMSO was added, samples were then incubated at RT. After 1 hour, 4µL of 6X SDS sample buffer was added to each reaction and samples were separated by SDS-PAGE. One gel was stained with Coomassie to verify similar amounts of loaded protein, while the other gel was used to transfer onto nitrocellulose membrane. Membrane was blocked with 5% nonfat dry milk in 20 mM Tris, 500 mM NaCl pH 8.0 (TBST) 1 hour at RT, then incubated overnight at 4℃ with 1:5,000 dilution of anti-thiophosphate ester antibody (Abcam ab92570). The membrane was washed 3x with TBST, incubated 1 hour at RT with 1:1000 dilution of goat anti-rabbit secondary antibody (Jackson ImmunoResearch HRP-GARIgG 111-035-003 in 1% milk), washed 3x with TBST before reaction with peroxidase substrate (Pierce 32209) and visualized with film.

Fluorescence microscopy

Vc strains were grown in LB plus antibiotic and inducer, if necessary, at 37°C until mid-log (OD_{600} = ~0.5). *Vc* were centrifuged at 7,000 rcf 2 min, and resuspended in 1x PBS. 1% agarose pad was placed gently on top of 5 µL of cells, and imaged with an Olympus BX43 microscope using ORCA-spark CMOS camera (Hamamatsu). sfgfp was detected using the U-FBNA BX3 filter cube (470-495 nm / 510-550 nm) and sfCherry was detected using the U-FGNA BX3 filter cube (540-550 nm / 575-625 nm). Fluorescent signal was quantified using Fiji (ImageJ), determining the fluorescent intensity along the medial axis of individual cells (Make Plot

function). Fluorescent intensity was plotted against the relative cell length using GraphPad Prism (cells analyzed = 100 per strain).

Mouse Intestinal colonization assay

Vc mutant strains were each mixed in a 1:1 ratio with the isogenic wild-type strain and then inoculated intragastrically into 5-day-old CD-1 suckling mice (approximately 10^6 mutant: 10^6 wildtype). Infected pups were placed in a humidified 30°C incubator. After 22 h, the mice were euthanized, and their small intestines were isolated and homogenized. The mutant/wildtype ratios were determined by plating dilutions on LB agar containing X-Gal (5-bromo-4-chloro-3-indolyl-D-galactopyranoside). The competitive index is given as the output ratio of mutant:wild type divided by the input ratio of mutant:wild type; Prism 5.0b was used for statistical analyses using the Student's paired t-test.

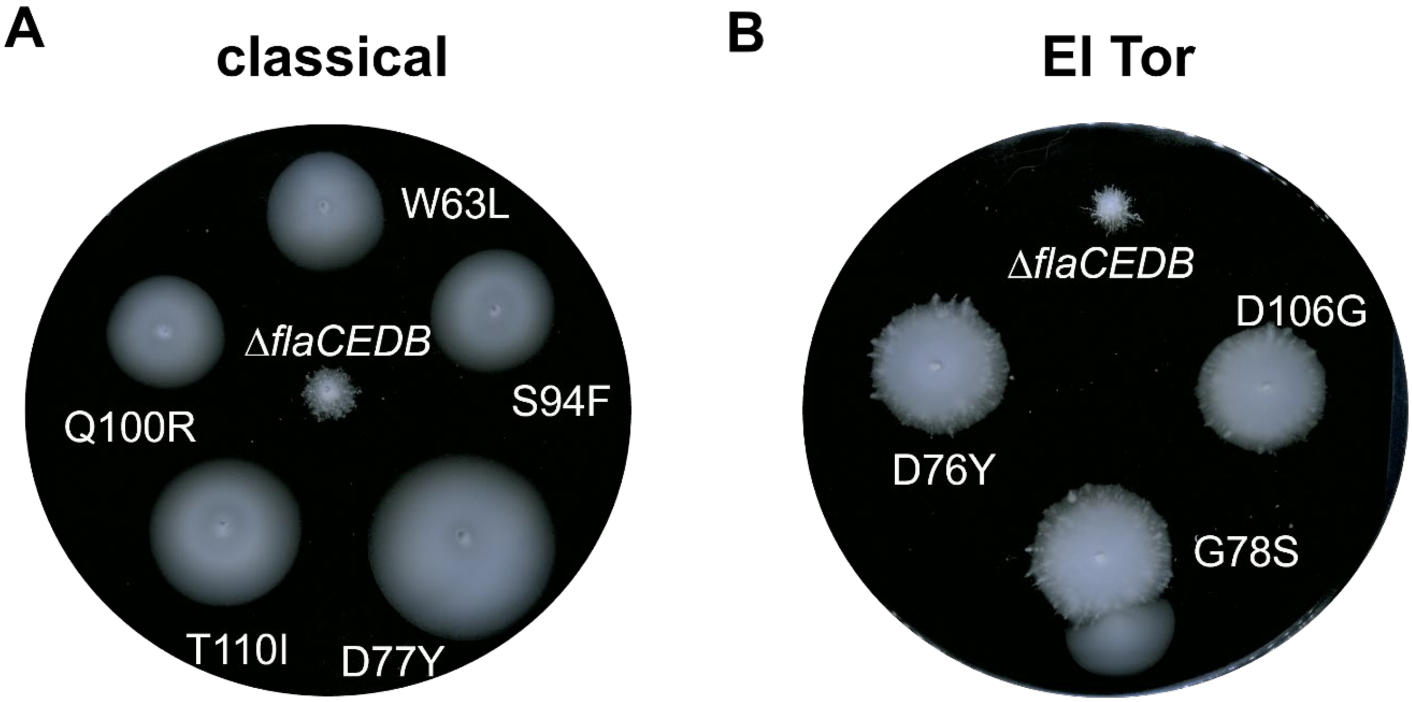

Fig S1. Motility of spontaneous PAS domain mutations in Δ*flaCEDB* in classical (O395; A) and El Tor (A1552; B) background. *V. cholerae* strains were inoculated into motility agar at 30°C; motility is visualized by swarm diameter. Strains shown (**A**) are KKV2530 (O395 Δ*flaCEDB*), KKV2570 (Δ*flaCEDB flrBW63L*), KKV2572 (Δ*flaCEDB flrBS94F*), KKV2575 (Δ*flaCEDB flrBD77Y*), KKV2576 (Δ*flaCEDB flrBT110I*) and KKV2578 (Δ*flaCEDB flrBQ100R*). Strains shown (**B**) are KKV3159 (A1552 Δ*flaCEDB*), KKV3169 (Δ*flaCEDB flrBD106G*), KKV3174 (Δ*flaCEDB flrBG78S*), and KKV3175 (Δ*flaCEDB flrBD76Y*).

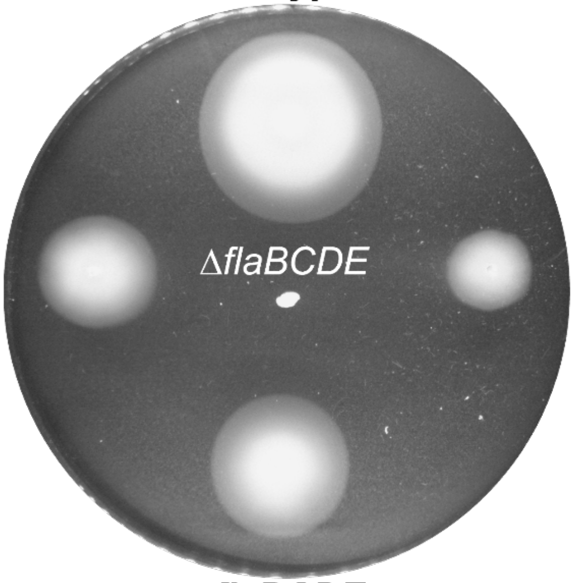

Fig S2: PAS mutations L36F and D76Y, or overexpression of FlaA, in Δ*flaCEDB* strain restore motility. *Vc* strains were inoculated into motility agar containing 0.1% arabinose at 30°C; motility is visualized by swarm diameter. Strains shown are KKV598 (wildtype O395), KKV2530 (Δ*flaCEDB*), KKV2530 carrying plasmid pKEK1948 (pBAD-flaA), KKV4091 (Δ*flaCEDB flrBL36F*) and KKV4089 (Δ*flaCEDB flrBD76Y*).

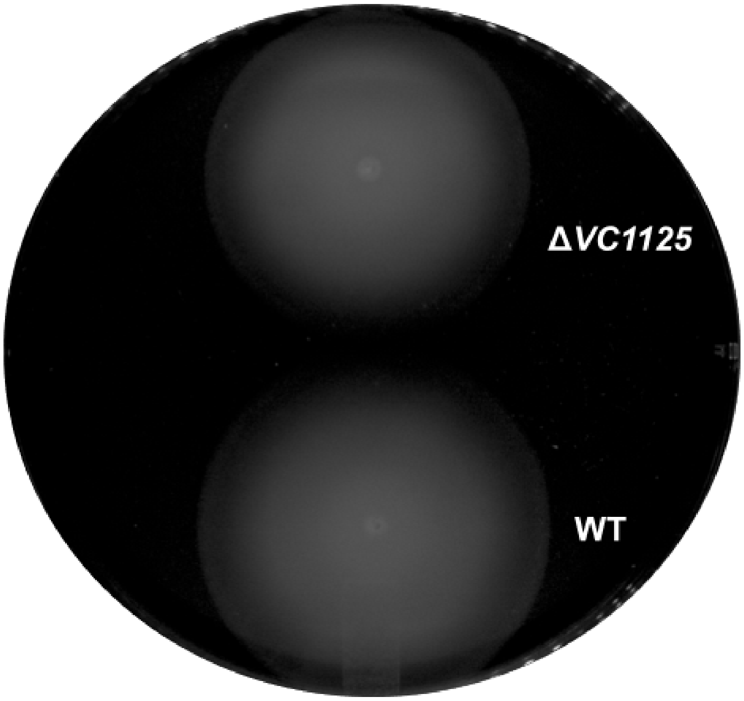

Figure S3. Motility of ΔVC1125. *Vc* strains were inoculated into motility agar at 30°C; motility is visualized by swarm diameter. Strains shown are KKV598 (wildtype O395) and KKV4407 (ΔVC1125).

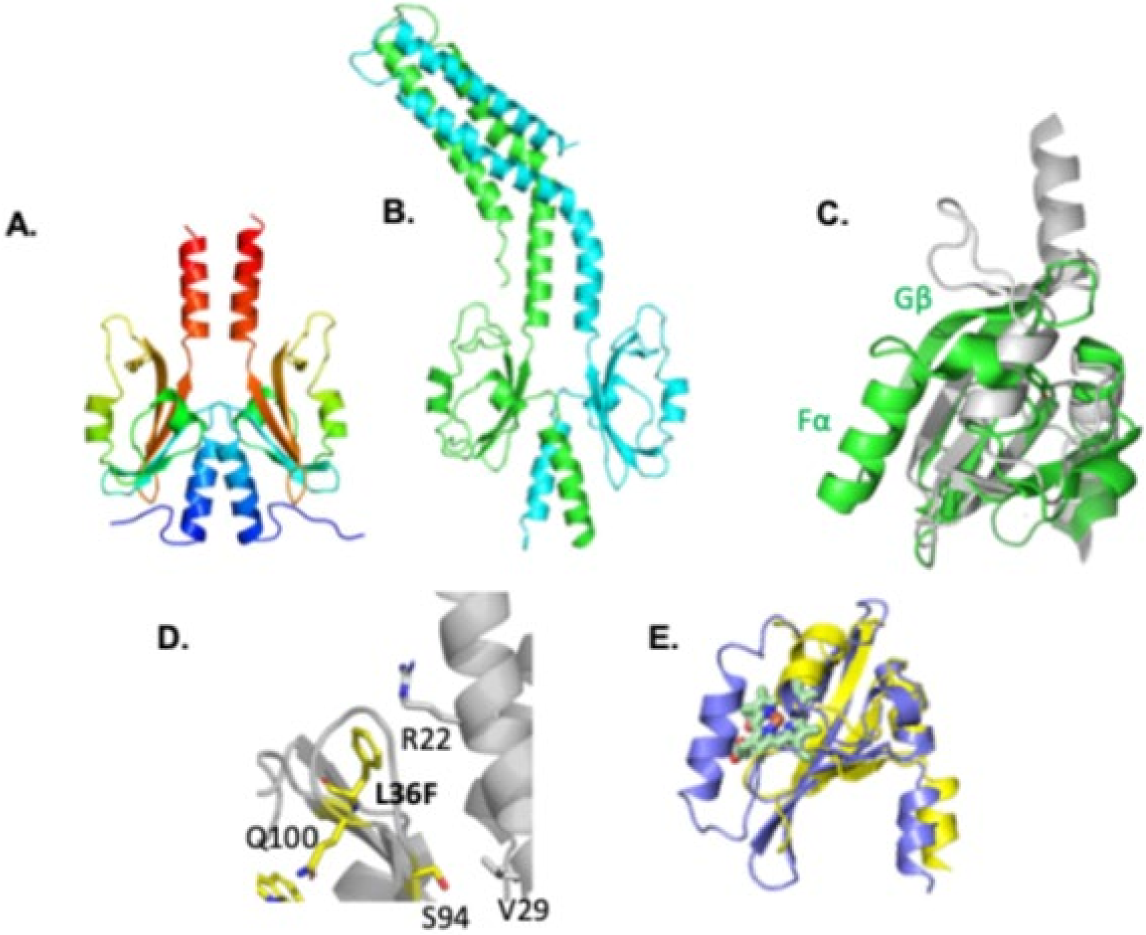

Figure S4. (A) The PAS domain of FlrB from *V. cholerae* (PDB code 7yrt) compared to **(B)** the dimer from our crystal structure (PDB code 9p6i) of FlrB N-terminal domain. The orientation of the structure in **B** is the same as that of **A**. **(C)**. Superposition of FlrB PAS (grey) with prototype Photoactive Yellow Protein (PYP; green) (PDB code 1nwz). Fα and Gβ are missing from FlrB PAS. **(D)**. Zoomed-in view shows that L36F substitution could alter interaction with R22. **(E).** Superposition of FlrB PAS from *V. cholerae* (PDB code 9p6i, yellow) with the heme-bound PAS domain from *E. coli* (PDB code 1v9z, purple). The alpha helix in the heme-bound PAS domain that directly interacts with heme is missing in *Vc* PAS.

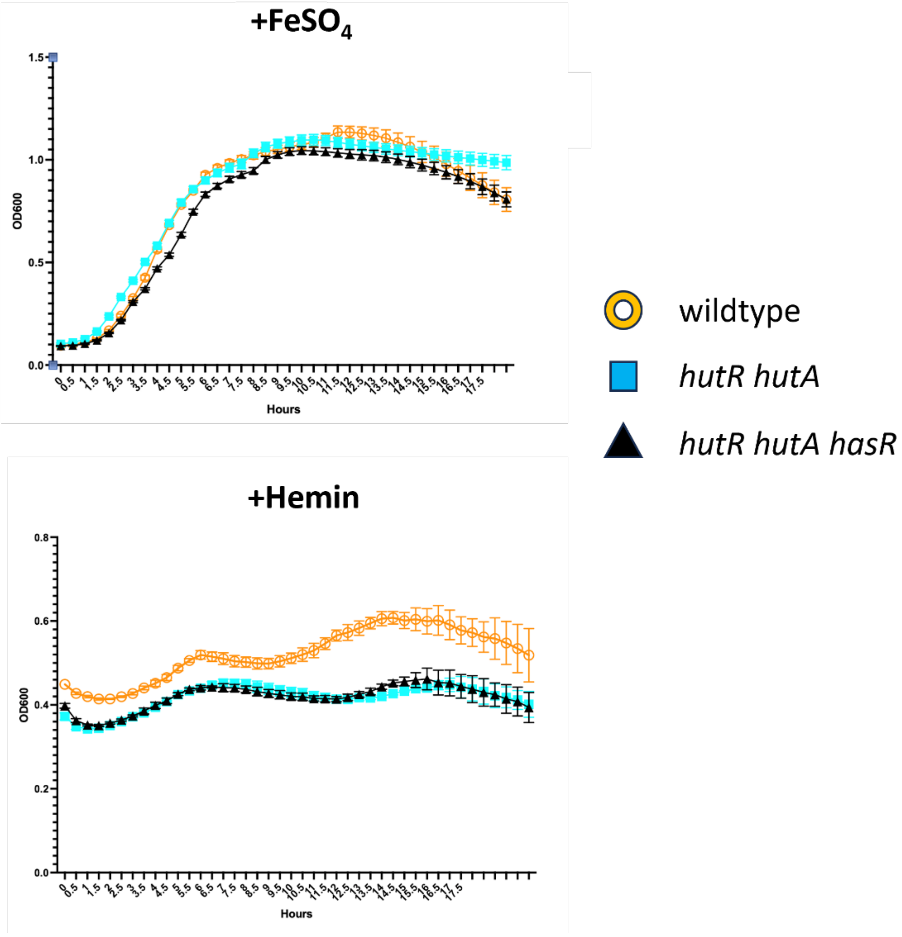

Figure S5. Vc heme transport mutants fail to grow using heme as iron source. *Vc* strains were grown at 37°C in LB media first depleted of iron by addition of 100 μg/ml ethylenediamine-di-(o-hydroxyphenylacetic acid) (EDDA), then supplemented with 40 μM ferrous sulfate (top) or 5 mM hemin (bottom), growth measured by OD_{600}. Strains shown are KKV598 (wildtype O395), KKV4047 (Δ*hutR* Δ*hutA*), and KKV4059 (Δ*hutR* Δ*hutA* Δ*hasR*).

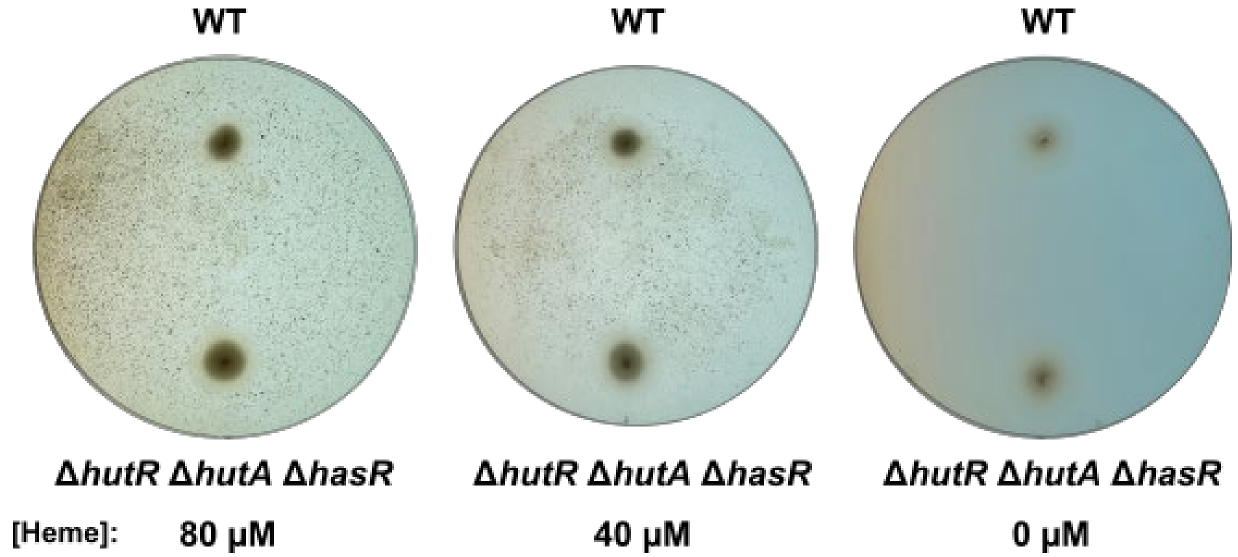

Figure S6. Vc heme transport mutant exhibits wildtype motility in the presence of heme. *Vc* strains were inoculated into motility agar containing 0, 40 μM or 80 μM hemin at 30°C; motility was visualized on light box because of dark coloration of media due to presence of hemin. Strains shown are KKV598 (wildtype O395) and KKV4059 (Δ*hutR* Δ*hutA* Δ*hasR*).

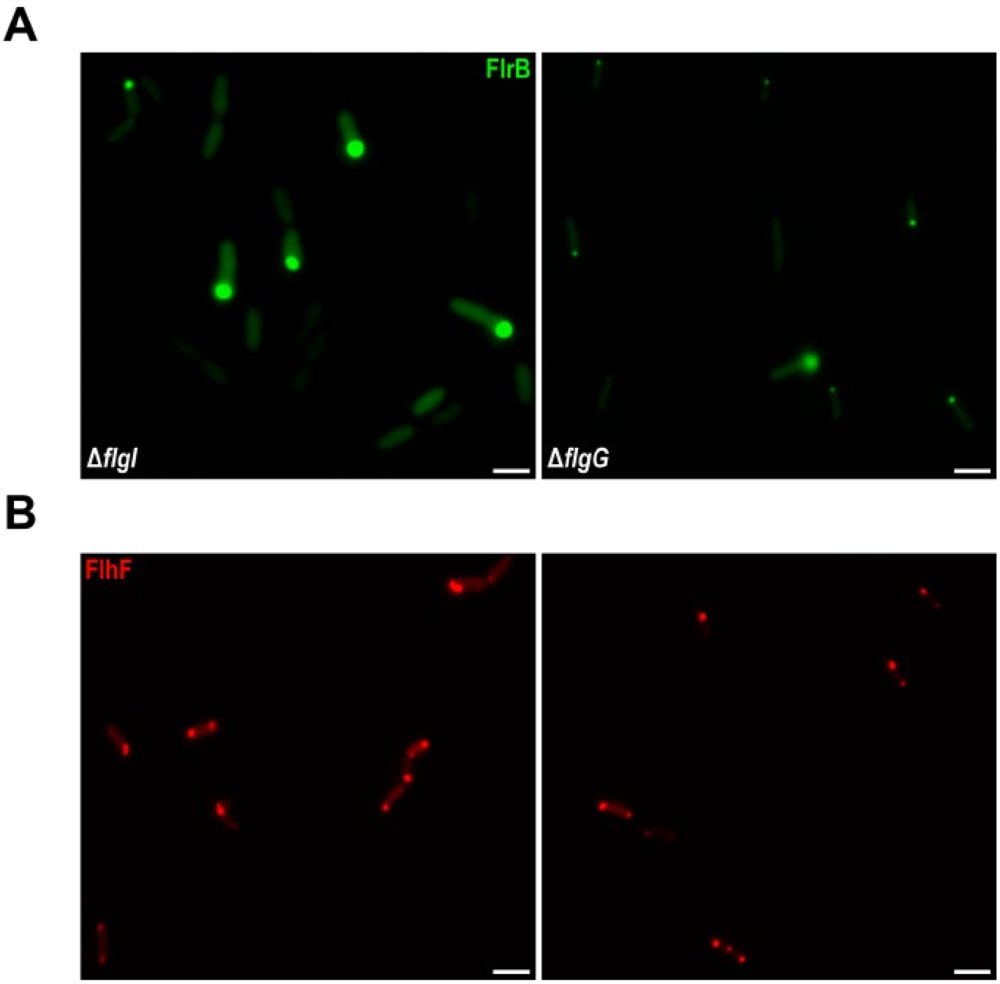

Figure S7 A. FlrB-sfgfp localizes to the pole independent of FlgG or FlgI. *Vc* strains KKV2772 (Δ*flgG*) or KKV2773 (Δ*flgI*) containing plasmid pKEK3494 (FlrB-sfgfp), were grown in the presence of 0.2% arabinose and observed by fluorescence microscopy. **B. FlhF-sfcherry localizes to the pole.** *V. cholerae* strain KKV1560 (Δ*flhF*) cells containing plasmid pKEK3590 (FlhF-sfcherry), were grown in the presence of 1 μg/ml Anhydro-tetracycline and observed by fluorescence microscopy. Scale bars = 1 μm

Table S1: FlrB Structure Data quality and refinement statistics.

**PDB Accession Code**	**9p6i**
**Data Collection**	
Space group	$P3_1$
Unit cell parameters (Å; º)	a = 86.21, b = 86.21, c=212.64;
	α = 90.00, β = 90.00, γ = 120.00
Wavelength	0.97848
Resolution range (Å)	30.00 - 2.75 (2.80 - 2.75)[a]
No. of reflections	45,970 (2,314)
R_{pim} (%)	3.4 (88.6)
$CC_{1/2}$ (%)	99.5 (63.4)
Completeness (%)	99.9 (100.0)
$\langle I/\sigma(I) \rangle$	23.9 (1.9)
Multiplicity	10.7 (10.9)
Wilson *B* factor	68.8
**Refinement**	
Resolution range (Å)	28.22 - 2.75 (2.82 - 2.75)
Completeness (%)	99.9 (99.7)
No. of reflections:	
Working set	41,253 (3,241)
Free R test set[b]	2,322 (156)
R_{work}/R_{free}, (%)	24.0/28.6 (30.5/38.0)
Protein chains/atoms	8/10,887
Ligand/Solvent atoms	213/57
Mean temperature factor ($Å^2$)	93.1
**Coordinate Deviations**	
R.m.s.d. bonds (Å)	0.002
R.m.s.d. angles (º)	0.845
**Ramachandran plot**	
Favored (%)	92.0
Allowed (%)	8.0
Outside allowed (%)	0.0

[a] Values in parentheses are for the outer shell.
[b] Free R value test size is 5.1%

Table S2: Oligonucleotides used in this study

primer	sequence
flrBp F FC	CGAGCTCGGTACCCGGcagccagagcctagccgttttag
flrB R FC	CTTGCATGCCTGCAGGcatcttcactctccagtctctgagtttg
flrB H135N F	cgtcattggcCAaccaagtgagaacgccactgtc
flrB H135N R	ctcacttggtTGgccaatgacgccaccatgcg
Universal 1	CCGGGTACCGAGCTCGAATTC
Universal 2	CCTGCAGGCATGCAAGCTT
DelflaEDB+AbR R	TTGAACCGCTTAGGGTCATGGCTGCTGCACTCCTTAC
DelflaEDB+AbR F	TTTTCTGCCATCAGTTTAACCCTTTGAGCGGTATTGAGC
Del flaC flank1 F	aattCgagctcggtacccggggatctgttcaaagcttcggttg
Del flaC flank1 R	GAActaCTCGAGcgccatGgtggtatctcctattgattttccgac
Del flaC flank2 F	cacCatggcgCTCGAGtagttcgagttgataggaaaaaaccc
Del flaC flank2 R	caagCttgcatgcctgcaggcgcaccagatttaggttctgg
Del flaC AbR F	TTTTCTGCCATCAGTTtagttcgagttgataggaaaaaaccc
Del flaC AbR R	TTGAACCGCTTAGGGTcatGgtggtatctcctattgattttcc
flhA F FC	CgagctcggtacccggCCCCTAGTGATGCACAAAGAGC
Del flhA R SalI	GACGATTAgtcgacCATAAATTTCTGGCTGTCACTGAAGGG
Del flhA F SalI	AATTTATGgtcgacTAATCGTCCGCACGTTTTTTAAAGACGG
flhA R FC	cttgcatgcctgcaggCATTTCCAAAGAGACACCCATGCG
Del flhA R AbR	GTCGACGGATCCCCGGAATCATAAATTTCTGGCTGTCACTGAAGGG
Del flhA F AbR	GAAGCAGCTCCAGCCTACATAATCGTCCGCACGTTTTTTAAAGACGG
ctxA flank1 F HiFi	gctcggtacccggggatccttgggaatgctgtcgggag
ctxA flank1 R	TTCTggatcccatataatgctccctttgtttaacagaaaaataattg
ctxA flank2 F	TATGggatccagaattaaggatgaattatgattaaattaaaatttg
ctxA flank2 R HiFi	Caagcttgcatgcctgcaggtacgcactaaggatgtgg
flrB NcoI F BsaI	ggctacggtctcccATGGCgagcagcgcagtgcaagag
flrB noBsaI R	aaagtgaagaAccatgttgctgggtaatgc
flrB noBsaI F	gcaacatggtTtcttcactttcgacttcttgacc
flrB fusion R SalI SphI BsaI	ggctacggtctccatgcctctccagtctctgagtttgagc
sfGFP F NcoI SphI GG BsaI	GgctacggtctcccatggcTgcatgcGGAGGAGGAGGAGGAATGAGC
sfGFP R SalI GG BsaI	ggctacggtctcgtcgacTCATTTGTAGAGCTCATCCATGCC
VC1125 F BbsI:	GgctacGAAGACggcccgGCGTTAGTGGCGATATCATTATGG
VC1125 SOE BHI R	CGTTTCAggatccTCTCATGGTCGATTCATTCCTATCAATTG
VC1125 SOE BHI F:	CATGAGAGgatccTGAAACGGTCTGGTATAAACCAAGTC
VC1125 BbsI R:	GgctacGAAGACgggcagTTCGCAACGTGATTGACGC
Sfcherry F NcoI SphI GG BsaI	GgctacggtctcccatggcTgcatgcGGAGGAGGAGGAGGAATG GAAGAAGATAATATGGCAATTATCAAGG

Sfcherry R SalI GG BsaI	ggctacggtctcgtcgacTCATGTAGAGTGACGTGCCTC
VcflhF F NcoI BsaI GG	GgctacggtctcCCATGAAAATAAAACGATTTTTTGCCAAGG
VCflhF R Sal ISphI BsaI	ggctacggtctcgtcgacCTAgcatgcGAATCTCTCTGAATCACTGGTCCAG

Table S3: Plasmids used in this study

Plasmid	Description	reference
pAMS4	*hasR*::*tmp* in pHM5 (sucrose-counterselectable suicide vector)	(29)
pBAD-FLP	Arabinose-inducible FLP expression plasmid	(7)
pCVD442	Sucrose-counterselectable suicide vector; AmpR	(30)
pFY5790	pGP704-hubP-sfgfp	F. Yildiz
pKEK83	Δ*flrB* in pCVD442	(31)
pKEK229	Sucrose-counterselectable suicide vector; AmpR	(6)
pKEK424	Δ*fliF* in pKEK229	(32)
pKEK516	Δ*flhF* in pKEK229	(33)
pKEK1854	Δ*flaEDB* in pKEK229	(34)
pKEK1948	pBAD-flaA	(34)
pKEK1994	Δ*flaC* in pKEK229	(34)
pKEK2013	Δ*flgG* in pKEK229	This study
pKEK2014	Δ*flgI* in pKEK229	This study
pKEK2094	Cloning vector; pUC origin; AmpR	(1)
pKEK2154	Δ*ctxA* in pKEK2200	This study
pKEK2158	*flrB* WT in pKEK2200	This study
pKEK2164	*flrB L36F* in pKEK2200	This study
pKEK2200	Sucrose-counterselectable suicide vector; CmR	(1)
pKEK2202	Sucrose-counterselectable suicide vector; AmpR	(1)
pKEK2222	Δ*flaC* in pKEK2202	This study

pKEK2227	Δ*flaEDB*::SpcR in pKEK229	This study
pKEK2270	pWKS30Tc*chapR*	(8)
pKEK2285	ptac-*tfoX*-*qstR*-*sacB*-oriT	(8)
pKEK2286	Δ*flaC*::SpcR in pKEK2202	This study
pKEK2377	*ΔflhA* in pKEK2094	This study
pKEK2388	Δ*flrB in pKEK2200*	This study
pKEK2395	*flrB H135N* in pKEK2200	This study
pKEK2402	ΔflhA::KanR in pKEK2094	This study
pKEK2405	*flrB L36F H135N* in pKEK2200	This study
pKEK2413	*flrB L36F H135N* in pKEK2200	This study
pKEK2442	flrB D76Y in pKEK2200	This study
pKEK2461	*flrB D76Y* H135N in pKEK2200	This study
pKEK2465	Δ*flhA* in pKEK2200	This study
pKEK2949	Sucrose-counterselectable suicide vector; CmR	This study
pKEK3093	pBAD CmR oriT w sfGFP	(5)
pKEK3370	pBAD-sfgfp	This study
pKEK3490	pBAD-flrB	This study
pKEK3493	pBAD-flrB-sfgfp	This study
pKEK3503	pBAD-flrB L36F	This study
pKEK3504	pBAD-flrB H135N	This study
pKEK3509	pBAD-flrB L36F-sfgfp	This study
pKEK3510	pBAD-flrB H135N-sfgfp	This study
pKEK3524	pBAD-sfcherry	This study
pKEK3590	pBAD- FlhF-sfcherry	This study
pKEK3607	pBAD-FlhF	This study
pKEK3642	Δ*VC1125* in pKEK2949	This study

Table S4: *V. cholerae* strains used in this study

Strain	Description	Reference/Source
O395	*V. cholerae* wildtype classical biotype	(35)
A1552	*V. cholerae* wildtype El Tor biotype	(36)
ARM313	*hutR::Cm*	(29)
ARM315	*hutA::Kan*	(29)
SAD033	*E7946 ΔVC1807::FRT-SpcR-FRT*	(4)
SAD034	*E7946 ΔVC1807::FRT-KanR-FRT*	(4)
KKV176	O395 *ΔflrB ΔlacZ*	(31)
KKV598	O395 *ΔlacZ*	(6)
KKV1247	O395 *ΔfliF ΔlacZ*	(37)
KKV1560	O395 *ΔflhF ΔlacZ*	(33)
KKV2530	O395 *ΔflaCEDB*	(34)
KKV2570	O395 *ΔflaCEDB; flrB W63L*	This study
KKV2572	O395 *ΔflaCEDB; flrB S94F*	This study
KKV2575	O395 *ΔflaCEDB; flrB D77Y*	This study
KKV2576	O395 *ΔflaCEDB; flrB T110I*	This study
KKV2578	O395 *ΔflaCEDB; flrB Q100R*	This study
KKV2579	O395 *ΔflaCEDB; flrB L36F*	This study
KKV2580	O395 *ΔflaCEDB; flrB G41D*	This study
KKV2772	O395 *ΔflgG; ΔlacZ*	This study
KKV2773	O395 *ΔflgI;ΔlacZ*	This study
KKV3000	A1552 *ΔctxA*	This study
KKV3077	A1552 *ΔflaEDB*::SpcR; *ΔctxA*	This study
KKV3104	A1552 *ΔflaEDB ΔctxA*	This study

KKV3153	A1552 *ΔflaC::SpcR; ΔflaEDB* Δ*ctxA*	This study
KKV3159	A1552 *ΔflaC; ΔflaEDB* Δ*ctxA*	This study
KKV3169	A1552 *ΔflaCEDB; flrB D106G;* Δ*ctxA*	This study
KKV3174	A1552 *ΔflaCEDB; flrB G78S;* Δ*ctxA*	This study
KKV3175	A1552 *ΔflaCEDB; flrB D76Y;* Δ*ctxA*	This study
KKV3288	O395 *flrB L36F;* Δ*lacZ*	This study
KKV3412	O395 Δ*flrB*; Δ*fliF*; Δ*lacZ*	This study
KKV3415	O395 *flrB H135N;* Δ*lacZ*	This study
KKV3436	O395 Δ*flhA::KanR;* Δ*flrB;*Δ*lacZ*	This study
KKV4027	O395 *hutA*::Kan Δ*lacZ*	This study
KKV4047	O395 *hutA*::Kan *hutR*::Cm Δ*lacZ*	This study
KKV4059	O395 *hasR*::tmp *hutA*::Kan *hutR*::Cm Δ*lacZ*	This study
KKV4071	O395 Δ*flrB ΔflaCEDB*	This study
KKV4075	O395 Δ*flrB ΔflaCEDB* Δ*lacZ*	This study
KKV4076	O395 *ΔflaCEDB* Δ*lacZ*	This study
KKV4089	O395 *ΔflaCEDB; flrB D76Y*	This study
KKV4090	O395 *ΔflaCEDB; flrB L36F H135N*	This study
KKV4091	O395 *ΔflaCEDB; flrB L36F*	This study

KKV4105	O395 Δ*fliF flrB D76Y* Δ*lacZ*	This study
KKV4106	O395 Δ*fliF flrB L36F H135N* Δ*lacZ*	This study
KKV4107	O395 Δ*fliF flrB L36F* Δ*lacZ*	This study
KKV4335	O395 Δ*flrB* Δ*flhF* Δ*lacZ*	This study
KKV4407	O395 Δ*VC1125* Δ*lacZ*	This study

Supplemental References

1. Mejia-Santana A, Lloyd CJ, Klose KE. 2021. New cloning vectors to facilitate quick allelic exchange in gram-negative bacteria. Biotechniques 70:116-119.
2. Garcia-Nafria J, Watson JF, Greger IH. 2016. IVA cloning: A single-tube universal cloning system exploiting bacterial In Vivo Assembly. Sci Rep 6:27459.
3. Horton RM, Hunt HD, Ho SN, Pullen JK, Pease LR. 1989. Engineering hybrid genes without the use of restriction enzymes: gene splicing by overlap extension. Gene 77:61-8.
4. Dalia AB, McDonough E, Camilli A. 2014. Multiplex genome editing by natural transformation. Proc Natl Acad Sci U S A 111:8937-42.
5. Armstrong ZR, Alonso J, Stanton V, Patel N, Zogaj X, Cocioba SS, Klose KE. 2025. Mobilizable shuttle vectors with fluorescent markers functional across different species of bacteria. Appl Environ Microbiol 91:e0004525.
6. Correa NE, Lauriano CM, McGee R, Klose KE. 2000. Phosphorylation of the flagellar regulatory protein FlrC is necessary for *Vibrio cholerae* motility and enhanced colonization. Mol Microbiol 35:743-755.
7. Scholz SA, Diao R, Wolfe MB, Fivenson EM, Lin XN, Freddolino PL. 2019. High-Resolution Mapping of the Escherichia coli Chromosome Reveals Positions of High and Low Transcription. Cell Syst 8:212-225 e9.
8. Lloyd CJ, Mejia-Santana A, Dalia TN, Dalia AB, Klose KE. 2021. Natural Transformation in a Classical-Biotype Vibrio cholerae Strain. Appl Environ Microbiol 87.
9. Fernandez NL, Hsueh BY, Nhu NTQ, Franklin JL, Dufour YS, Waters CM. 2020. Vibrio cholerae adapts to sessile and motile lifestyles by cyclic di-GMP regulation of cell shape. Proc Natl Acad Sci U S A 117:29046-29054.
10. Afgan E, Nekrutenko A, Grüning BA, Blankenberg D, Goecks J, Schatz MC, Ostrovsky AE, Mahmoud A, Lonie AJ, Syme A, Fouilloux A, Bretaudeau A, Nekrutenko A, Kumar A, Eschenlauer AC, Desanto AD, Guerler A, Serrano-Solano B, Batut B, Grüning BA, Langhorst BW, Carr B, Raubenolt BA, Hyde CJ, Bromhead CJ, Barnett CB, Royaux C, Gallardo C, Blankenberg D, Fornika DJ, Baker D, Bouvier D, Clements D, De Lima Morais DA, Tabernero DL, Lariviere D, Nasr E, Afgan E, Zambelli F, Heyl F, Psomopoulos F, Coppens F, Price GR, Cuccuru G, Corguillé GL, Von Kuster G, Akbulut GG, Rasche H, Hotz H-R, Eguinoa I, et al. 2022. The Galaxy platform for accessible, reproducible and collaborative biomedical analyses: 2022 update. Nucleic Acids Research 50:W345-W351.
11. Prjibelski A, Antipov D, Meleshko D, Lapidus A, Korobeynikov A. 2020. Using SPAdes De Novo Assembler. Current Protocols in Bioinformatics 70.
12. Mikheenko A, Prjibelski A, Saveliev V, Antipov D, Gurevich A. 2018. Versatile genome assembly evaluation with QUAST-LG. Bioinformatics 34:i142-i150.
13. Tatusova T, Dicuccio M, Badretdin A, Chetvernin V, Nawrocki EP, Zaslavsky L, Lomsadze A, Pruitt KD, Borodovsky M, Ostell J. 2016. NCBI prokaryotic genome annotation pipeline. Nucleic Acids Research 44:6614-6624.
14. Robinson MD, Oshlack A. 2010. A scaling normalization method for differential expression analysis of RNA-seq data. Genome Biol 11:R25.
15. Robinson MD, McCarthy DJ, Smyth GK. 2010. edgeR: a Bioconductor package for differential expression analysis of digital gene expression data. Bioinformatics 26:139-40.
16. McCarthy DJ, Chen Y, Smyth GK. 2012. Differential expression analysis of multifactor RNA-Seq experiments with respect to biological variation. Nucleic Acids Res 40:4288-97.
17. Benjamini Y, Hochberg Y. 2018. Controlling the False Discovery Rate: A Practical and Powerful Approach to Multiple Testing. Journal of the Royal Statistical Society: Series B (Methodological) 57:289-300.
18. Kanehisa M, Furumichi M, Sato Y, Matsuura Y, Ishiguro-Watanabe M. 2025. KEGG: biological systems database as a model of the real world. Nucleic Acids Res 53:D672-D677.
19. Bu D, Luo H, Huo P, Wang Z, Zhang S, He Z, Wu Y, Zhao L, Liu J, Guo J, Fang S, Cao W, Yi L, Zhao Y, Kong L. 2021. KOBAS-i: intelligent prioritization and exploratory visualization of biological functions for gene enrichment analysis. Nucleic Acids Res 49:W317-W325.
20. Stols L, Gu M, Dieckman L, Raffen R, Collart FR, Donnelly MI. 2002. A new vector for high-throughput, ligation-independent cloning encoding a tobacco etch virus protease cleavage site. Protein Expr Purif 25:8-15.
21. Shuvalova L. 2014. Parallel protein purification. Methods Mol Biol 1140:137-43.

22. Minor W, Cymborowski M, Otwinowski Z, Chruszcz M. 2006. HKL-3000: the integration of data reduction and structure solution--from diffraction images to an initial model in minutes. Acta Crystallogr D Biol Crystallogr 62:859-66.
23. Murshudov GN, Skubak P, Lebedev AA, Pannu NS, Steiner RA, Nicholls RA, Winn MD, Long F, Vagin AA. 2011. REFMAC5 for the refinement of macromolecular crystal structures. Acta Crystallogr D Biol Crystallogr 67:355-67.
24. Emsley P, Lohkamp B, Scott WG, Cowtan K. 2010. Features and development of Coot. Acta Crystallogr D Biol Crystallogr 66:486-501.
25. Cohen SX, Ben Jelloul M, Long F, Vagin A, Knipscheer P, Lebbink J, Sixma TK, Lamzin VS, Murshudov GN, Perrakis A. 2008. ARP/wARP and molecular replacement: the next generation. Acta Crystallogr D Biol Crystallogr 64:49-60.
26. Painter J, Merritt EA. 2006. TLSMD web server for the generation of multi-group TLS models. J Appl Cryst 39:109-111.
27. Chen VB, Arendall WB, 3rd, Headd JJ, Keedy DA, Immormino RM, Kapral GJ, Murray LW, Richardson JS, Richardson DC. 2010. MolProbity: all-atom structure validation for macromolecular crystallography. Acta Crystallogr D Biol Crystallogr 66:12-21.
28. Carlson HK, Plate L, Price MS, Allen JJ, Shokat KM, Marletta MA. 2010. Use of a semisynthetic epitope to probe histidine kinase activity and regulation. Anal Biochem 397:139-43.
29. Mey AR, Payne SM. 2001. Haem utilization in Vibrio cholerae involves multiple TonB-dependent haem receptors. Mol Microbiol 42:835-49.
30. Donnenberg MS, Kaper JB. 1991. Construction of an *eae* deletion mutant of enteropathogenic *Escherichia coli* by using a positive-selection suicide vector. Infect Immun 59:4310-4317.
31. Klose KE, Mekalanos JJ. 1998. Distinct Roles of an Alternative Sigma Factor During Both Free-Swimming and Colonizing Phases of the *Vibrio cholerae* Pathogenic Cycle. Mol Microbiol 28:501-520.
32. Lauriano CM, Ghosh C, Correa NE, Klose KE. 2004. The Sodium-driven Flagellar Motor controls Exopolysaccharide Expression in *Vibrio cholerae*. submitted for publication.
33. Correa NE, Peng F, Klose KE. 2005. Roles of the regulatory proteins FlhF and FlhG in the Vibrio cholerae flagellar transcription hierarchy. J Bacteriol 187:6324-6332.
34. Echazarreta MA, Kepple JL, Yen LH, Chen Y, Klose KE. 2018. A Critical Region in the FlaA Flagellin Facilitates Filament Formation of the Vibrio cholerae Flagellum. J Bacteriol 200.
35. Mekalanos JJ, Collier RJ, Romig WR. 1979. Enzymic activity of cholera toxin. II. Relationships to proteolytic processing, disulfide bond reduction, and subunit composition. J Biol Chem 254:5855-5861.
36. Yildiz FH, Schoolnik GK. 1998. Role of rpoS in Stress Survival and Virulence of Vibrio cholerae. J Bacteriol 180:773-784.
37. Correa NE, Barker JR, Klose KE. 2004. The Vibrio cholerae FlgM homologue is an anti-sigma28 factor that is secreted through the polar sheathed flagellum. J Bacteriol 186:4613-4619.
